# Scaling-up and future sustainability of a national reproductive genetic carrier screening program

**DOI:** 10.1038/s41525-023-00357-w

**Published:** 2023-07-31

**Authors:** Zoe Fehlberg, Stephanie Best, Janet C. Long, Tahlia Theodorou, Catherine Pope, Peter Hibbert, Sharon Williams, Lucinda Freeman, Sarah Righetti, Alison D. Archibald, Jeffrey Braithwaite

**Affiliations:** 1 Australian Institute of Heath Innovation, Macquarie University, Sydney, Australia; 2 Australian Genomics Health Alliance, Melbourne, Australia; 3 Murdoch Children’s Research Institute, Melbourne, Australia; 4 Department of Health Services Research, Peter MacCallum Cancer Centre, Melbourne, Australia; 5 Victorian Comprehensive Cancer Centre, Melbourne, Australia; 6 Sir Peter MacCallum Cancer Centre Dept of Oncology, University of Melbourne, Melbourne, Australia; 7 Nuffield Department of Primary Care Health Sciences, University of Oxford, Oxford, UK; 8 IIMPACT in Health, Allied Health and Human Performance, University of South Australia, Adelaide, Australia; 9 School of Health & Social Care, Swansea University, Swansea, Wales UK; 10 School of Women’s and Children’s Health, University of New South Wales, Sydney, Australia; 11 Graduate School of Health, University of Technology Sydney, Sydney, Australia; 12 Centre for Clinical Genetics, Sydney Children’s Hospital Network, Sydney, Australia; 13 Victorian Clinical Genetics Services, Murdoch Children’s Research Institute, Melbourne, Australia; 14 Department of Paediatrics, The University of Melbourne, Melbourne, Australia

**Keywords:** Genetics research, Population screening

## Abstract

An understanding of factors influencing implementation is essential to realise the benefits of population-based reproductive genetic carrier screening programs. The aim of this study was to synthesise data collected during the Australian Reproductive Genetic Carrier Screening Project (Mackenzie’s Mission) to track how priorities shifted over time and identify important factors during scaling-up and for sustainment. We used a multi-method qualitative approach to integrate longitudinal project data collected from 10 project committees with 16 semi-structured interviews conducted with study team members. Both datasets were analysed using the Consolidated Framework for Implementation Research (CFIR) to identify constructs of interest within early, mid-point, and future implementation phases. Several CFIR constructs were present across implementation. The complexity of implementation presented challenges that were overcome through a quality-designed and packaged product, formal and informal networks and communication, and access to knowledge and information. Addressing the diverse consumer needs through resources and increasing community and non-genetic speciality engagement remained a priority throughout and for future sustainment. Going forward, further addressing program complexities and securing funding were emphasised. By applying an implementation framework, findings from this study may be useful for future effort towards building and/or sustaining reproductive genetic carrier screening programs.

## Introduction

Interest in population-based reproductive genetic carrier screening (RGCS) programs is mounting as technology rapidly advances and costs fall. With ~1–2% of couples at an increased chance (usually 1 in 4) of having children with a severe recessive genetic condition^1^, professional bodies state information on RGCS should be offered to all individuals and couples pre-conception or in early pregnancy e.g.^2,3^,. Internationally, RGCS is predominantly accessed directly from commercial providers^4^ however, commercial provision raises concerns. Access is limited to those who know about RGCS and can afford it, the clinical validity of the screening test is less apparent—especially which genes are included, and there is variation in the quality and amount of pre- and post- test information and support provided^4–6^. In response, some countries, have begun implementing e.g., Israel^7^, or investigating e.g., the Netherlands’^8^ public RGCS programs.

In Australia, the federal government-funded research study, Australian Reproductive Genetic Carrier Screening Program, or “Mackenzie’s Mission”, provided thousands of Australian couples who were planning or in early pregnancy, access to free screening for 1,300 genes associated with around 750 conditions. The included genes were identified from 23 published commercial gene lists and reviewed by a team of clinical geneticists overseen by a multidisciplinary committee including genetic counsellors, an ethicist, a parent of a child with a genetic condition, and scientists. Gene selection criteria were the condition should be life-limiting or disabling with childhood onset and/or be one for which early diagnosis and intervention would substantially change outcomes and had strong evidence for gene-phenotype relationship. The gene list was continually reviewed and updated considering new information. Further details are reported elsewhere^9^. Mackenzie’s Mission was structured to enable the development and operationalisation of a national RGCS program and associated research components. Using couple-based screening, reproductive couples were invited to take part in Mackenzie’s Mission by a recruiting health care professional (HCP), typically General Practitioners (Family Physicians). Once invited, couples, including those using known donors, could access an online portal using a unique access code, complete education, use a decision aid if they wished, consent or decline, provide cheek swab samples via post and receive their results either online for ‘low chance’ results or via a study genetic counsellor if found to be at ‘increased chance’. Further study details are described elsewhere^10^.

Notwithstanding international interest, the reality of implementing population RGCS programs using structured implementation approaches is not well investigated. Although not commonplace in genomic medicine^11^, implementation science frameworks, when used, offer an understanding and explanation of how and why implementation efforts succeed or not^12^. By investigating factors influencing implementation across the implementation timeline and different contexts, frameworks can augment learnings, standardise terminology, and improve scaling-up and mainstreaming of services^13^. The Consolidated Framework for Implementation Research (CFIR) offers a comprehensive structure to identify and organise determinants that may influence implementation whilst providing a taxonomy for future efforts^14^. The CFIR situates 39 constructs derived from existing theories into five domains (Intervention Characteristics; Outer Setting; Inner Setting; Characteristics of Individuals; and Process). Here we apply the CFIR to track how priorities shift over time, identify factors influencing implementation during the scaling-up of a RGCS program, and outline factors that are likely to be important for sustainment going forward.

## Results

### Participants

In total, ISQs were collected at 345 meetings. The number of responses varied between committees depending on meeting frequency (between weekly and quarterly).

From the 20 operational staff identified, 16 responded to the invitation and were interviewed. Participants were representative of all Australian jurisdictions and included five clinical or laboratory study/state leads “Lead” (31%); six state coordinators “Coordination” (37.5%); two study genetic counsellors “Study GC” (12.5%); two laboratory scientists “Laboratory” (12.5%); and one senior member of a participating organisation “Snr Member” (5%). For identification in this study, participants’ primary operational role was selected although many had secondary responsibilities (i.e., state coordinator and study genetic counsellor).

### Findings by implementation phase and CFIR domains and constructs

Table 1 provides an overview of the CFIR domains identified (shaded) at the three implementation phases by dataset. Below, constructs of interest **(in bold)** are described within each CFIR domain and implementation phase. The integrated dataset is presented in Supplementary Table 4 and includes exemplar ISQs and interview quotes. CFIR constructs were not considered static and were able to evolve over the project timeline.

**Table 1:**
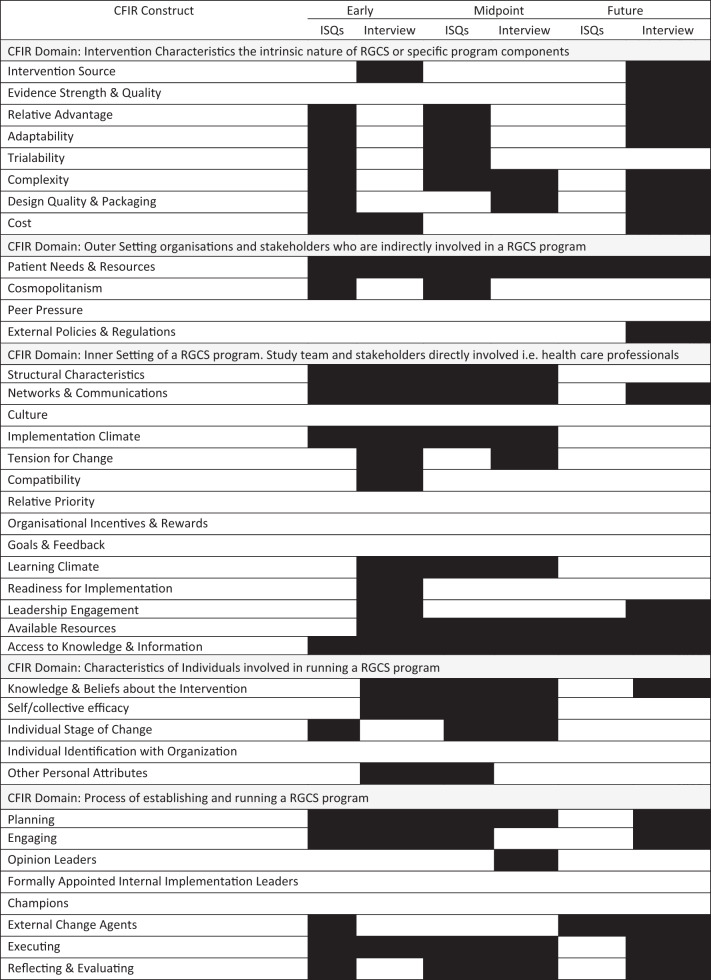
Consolidated Framework Implementation Research (CFIR) constructs by dataset and implementation phase.

Key: Shaded denotes the identification of the CFIR constructs within the data. Abbreviations: *CFIR* Consolidated Framework for Implementation Research, *ISQs* Implementation Science Questions, *RGCS* Reproductive Genetic Carrier Screening.

### Early implementation findings

#### Intervention characteristics

Present in the ISQs, was the **complexity** in scale and the intricacy of developing a clinically valid and useful gene list ‘*Preparing a gene list is a huge and somewhat daunting task*’ (Executive Committee). The need to **trial** and **adapt** components was acknowledged, especially to meet the needs of different contexts, improve accessibility, and respond to external factors such as the SARS-COV-2 pandemic. Whilst early efforts to **design and package** the program were considered successful, interview data indicated that in-house expertise was not fully recognised. As one participant put it:

*‘…we could have taken more advantage of the expertise we had [within the team] … it’s actually the little things that make a big difference when implementing a program … what do your test kits need to be like? And how do you make it really simple or straightforward for your target population? Sometimes we think too much about the higher-level stuff and we don’t give enough attention to the details.’* (Coordination 03)

#### Outer setting

ISQs and interviews showed how early **patient needs** were identified, **and resources** developed (e.g., a protocol to follow-up with couples with an increased psychosocial risk). ISQs indicate how the project utilised **cosmopolitanism** (the degree to which organisations/teams are networked) with external bodies to progress the development of quality study materials, e.g., ‘*The level of engagement in the Delphi group in designing the Decision Aid*’ (Psychosocial and Epidemiology Committee).

#### Inner setting

The project’s **structural characteristics** included the benefit of housing the project within established organisations. Some workforce skills were lacking and hindered project development but, once acquired, progress improved ‘*Now that [the] senior scientist [is] on board things are moving*’ (Laboratory Committee). **Networks and communication** was a dominant construct with fluctuations in the perceived quality of communication between the numerous teams and locations, posing challenges to keeping relevant parties informed and unified ‘*[it is] sometimes difficult to disseminate drafts and encourage collaboration*’ (Education and Engagement Committee). The **implementation climate** within the study team indicated their prior experience and perceived **tension for change**, meant there was a clear **readiness for implementation**. Especially noted was the strong **leadership engagement**, and the adequate **resources made available**, although how the resources were allocated was debated, with funding to incentivise HCPs engagement considered less justified in comparison to the operational needs of the program. Whereas the **implementation climate** of the broader community centred on the supportiveness of the genetics’ community (laboratory and clinical) and the mixed receptivity of the HCPs whose role it was to offer RGCS to their patients. Whilst ISQs reported HCP enthusiasm, upon reflection, interview participants noted HCPs’ prior experience of and perceived **compatibility** towards incorporating RGCS into their practice affected HCP engagement and ease of implementation:

*‘It was quite hard to get it started here … even though the project had been running elsewhere. There wasn’t a really good general knowledge out there, certainly not in General Practices, or obstetric practices … and the number of people already offering RGCS was extremely limited.’* (Lead 04)

#### Process

There was considerable overlap between **planning,** **engaging**, and **executing**. The ISQs focus on planning for the ‘soft launch’ whereas interviews reflected on areas of success (drawing on pilot studies) and improvement (e.g., greater involvement of Aboriginal and Torres Strait Islander communities at the outset). Interviews and ISQs noted the success of different HCP **engagement strategies**. ISQs tracked challenges and how well **plans** were being **executed** ‘*challenging to meet deadlines—a lot of material that needs to be put together all happening in parallel*’ (Education and Engagement Committee) and achievements *‘[Consumer] instruction sheet gone really well by increasing compliance rate substantially*’ (Laboratory Committee).

### Midpoint implementation findings

#### Intervention characteristics

Interview participants highly regarded the **design, quality and packaging** of the study materials. The streamlined approach was appealing to HCPs and couples, generating positive engagement with the program. Further, careful consideration during the **design** of sample collection instructions reduced laboratory and clinical workloads by delivering lower-than-expected re-collection rates. Program components that were **adaptable** to external factors (SARS-COV-2 pandemic) and project needs (e.g., GC workload) were welcomed, ‘*COVID-19 has impacted the way we deliver education in some positive ways (flexibility, lower resource use, geographic spread, ‘catch-up’ sessions)*’ (Education and Engagement Committee). In contrast, components that were **not adaptable** (e.g., collecting family history information) were considered burdensome throughout. ISQs and interviews indicated ongoing **complexities** associated with **trialling** implementation in a real-world setting. Noted were the difficulties of de-implementing or removing genes considering new evidence, and the required ‘*flexibility* ***[adaptability]*** *in the gene panel and the value of road-testing* ***[trialling]*** *the panel thoroughly*’ (Variant Review Committee). Clinical workloads were also impacted by the **complex** counselling required for ‘increased chance’ couples who already had children or where genes varied in phenotype.

#### Outer setting

ISQs tracked the progress of developing **resources to address patient needs** to increase the accessibility of screening, including re-designing the waiting-room poster in collaboration with Aboriginal health services. One committee was surprised at the challenges associated with addressing accessibility *‘The length of time it has taken Mackenzie’s Mission to address accessibility of the project in terms of languages other than English, and in format for sight impaired individuals.’* (Engagement Committee). Feedback and monitoring of **resources** were considered valuable to continuing development ‘*The amount of time [it takes] to recruit a non-English speaking couple shows the benefit of having translated materials*’ (Operational team).

#### Inner setting

Interview participants noted the importance of **networks and communication** to facilitate operating at a national scale. The program model fostered new relationships between genetics services and HCPs which interview participants felt would strengthen future service delivery. Formal (via recurrent meetings) and informal (via strong working relationships) **communication** between clinical and lab staff was considered by interview participants to provide **access to knowledge and information**. Effective communication was perceived to enable national collaboration, easy flow of information, and consensus on decisions to implement a consistent and high-quality national program. In particular, the weekly variant review committee was highly valued, as one participant put it.

‘*The thing about the review committee for Mackenzie’s Mission is we have 1300 genes across all sorts of different diseases. Nobody has all that expertise … And we have 30 to 40 people every week from around the country. And so far, no matter what gene has come up, one of the clinical geneticists has seen a patient with that condition*.’ (Lead 02)

#### Characteristics of individuals

ISQs reported increases in **self or collective efficacy** as the program scaled-up and **attributes** such as ‘innovation’ were celebrated in overcoming ‘curve balls’ (e.g., the SARS-COV-2 pandemic).

#### Process

**Executing plans** focused on striking the balance between opening screening to more people and laboratory capacity. Interviews and ISQs captured how despite being established as a couple-based screening program ‘*1300 genes has been very ambitious…We thought that most* *couples* *would have no variants that need looking at and would be able to be whipped through but in fact that’s the exception rather than the rule and so that has meant that the lab side has been harder than we thought*’ (Lead 01) and ‘*low number of cases that have no variants to review (i.e., there is a lot of analysis)*’ (State team) increased laboratory workloads. As such, longer turn-around times delayed opening the study to couples in early pregnancy and complicated clinical workloads as more couples became pregnant whilst waiting for results. ISQs tracked how plans to increase accessibility changed and one interview participant reflected upon the benefit of flexibility and **adaptability** to overcome the challenges of working in the real world:

‘*It’s hard to anticipate what is actually going to happen… and we could come up with as many plans as we like while everything is in theory and then we got to the real world … But the good thing about Mackenzie’s Mission was that it was so flexible, and we could change things as we moved through*.’ (Study GC 01)

### Future implementation findings

#### Intervention characteristics

Interview participants considered a reproductive couple-based versus an individual approach delivered in primary care had **relative advantage** for future efforts as it reduces patient anxiety, program **costs**, and genetic service **resources**. Also discussed were the implications of funding models:

*‘In an ideal world it would be a national program. Because a national program would be able to deliver consistency in terms of how it is delivered, what we are screening for, and address some of these equity of access issues. In reality, it is going to be a [publicly] funded test, which means you are purely funding the test and not the service that sits around it … [which] is probably 80% of the whole thing.’* (Coordination 03)

**Complexity** remained a dominant construct. Interview participants discussed simplifying the program by refinement of the gene list to ensure it aligns with screening principles, address concerns around informed consent, and deliver consistency in reporting of results, especially in the absence of a weekly variant review committee.

*‘… it’s going to be a complex thing trying to make the process, if it was available to all, simple yet comprehensive and that is going to be a big challenge going forward.’* (Coordination 04)

Again, having **quality consumer resources** was perceived as key to future success and overcoming complexity.

*‘… the front end is fantastic … we’ve got to minimise the genetic counsellor requirement at the front end by having fantastic resources for people to get the info*.’ (Lead 01)

Further, reduced complexity was perceived as less resource intensive, allowing resources to be directed to addressing laboratory and clinical workforce capacity (e.g., robotics and well-**designed and packaged consumer resources**). One participant noted the success of the program model tested during the study and its use for future healthcare delivery.

*‘The project was designed to be accidentally COVID proof. With everything happening online and with the postal kits, it’s really proved that model. And now with the way that healthcare has been influenced by COVID, it will be more and more acceptable for people to do something online and to not necessarily have face-to-face contact.’* (Coordination 01)

When thinking implementation at scale, **cost** became a salient theme, again a more **complex** program would increase **costs**.

‘*The more grey you have, the more resources you have to put into sorting it out and the more expensive the program becomes. That’s why when you’re doing something at scale you have to keep it really simple*.’ (Coordination 03)

#### Outer setting

Interview participants considered **patient needs** a future priority so that all individuals who wish to access screening are aware and able to access it, especially for Australia’s diverse cultural population including Aboriginal and Torres Strait Islander People. External **policies** that address financial barriers were perceived as a ‘top down’ approach to influence HCPs and consumer awareness and behaviour towards RGCS.

#### Inner setting

**Resourcing** for future RGCS was a concern raised in interviews with participants acknowledging the amount of human resourcing required, as one participant put it:

*‘To run a national RGCS program you are going to need staff. You are going to need enough laboratory staff to be able to provide screening in an appropriate timeframe. You are going to need dedicated genetic counsellors … who are not just involved in recruiting but in all the aspect around people, putting family history on forms, all that sort of thing*.’ (Lead 04)

Interview participants raised that future efforts should utilise the expertise of experienced individuals and professionals, such as bioinformaticians, who were considered invaluable during program development. ‘*Get a really good bioinformatician, they are worth their weight in gold, and they really understand what you’re trying to achieve but also the technical side*.’ (Coordination 01).

#### Process

Discussion around **engagement** shifted to reaching more HCPs, including finding a streamlined way to offer HCP education. Suggested were ‘*webinars that are held every so often rather than ad hoc when someone is interested’* (Coordination 02) and building education into continuing professional development courses. Increased public awareness and a focus on cultural safety were discussed as key areas for future efforts, with school programs and engagement of Aboriginal health care workers suggested as avenues for RGCS education. Participants acknowledged the influence of **external change agents** such as government investment in RGCS as a critical factor to secure funding and sustainability.

## Discussion

By applying the CFIR to a multi-method investigation, this study identifies key factors influencing the implementation of population RGCS. By identifying relationships between CFIR domains^15^, a coherent and complete picture can be assembled with the drivers behind determinants established. By categorising our findings within three distinct implementation phases, we tracked how factors re-align with shifts in project priorities. Previous research has centred on important considerations and challenges for implementing population RGCS^4,16–18^. We extend this work by linking scalability and sustainability priorities in the ‘real-world’, to implementation determinants. Our longitudinal approach addresses the persistent implementation gap between the availability of the technology and application in practice^19,20^. Below we discuss several overarching **CFIR coding** and draw on The Expert Recommendation for Implementing Change (ERIC)^21^ as a tool to align CFIR constructs with evidence informed implementation strategies^22^ and project examples (Table 2). Maintaining the purpose of this study as exploratory, application of the ERIC is limited to hypothesis generation about which strategies are likely to be important for implementing RGCS. Future endeavours can utilise the information to develop targeted and local context specific strategies.

**Table 2:** Prominent CFIR^14^ constructs linked with ERIC^22^ strategies and project learnings.

Period	CFIR construct	Highest rated ERIC strategies	Project learnings
Early	Design quality and packaging	• Promote adaptability • Obtain and use patients/consumers and family feedback • Develop educational materials	Resources: that are well-designed and streamline processes improve consumer and health care professional engagement and supports informed decision making (e.g., online portal housing education material and a decision aid)
	Implementation Climate	• Assess for readiness and identify barriers and facilitators • Alter incentive/allowance structures • Identify and prepare champions	Engaging: non-genetic health care professionals requires a nuanced and context driven approach to accommodate varying skill and knowledge levels, confidence, and motivation
	Complexity	• Develop a formal implementation blueprint • Promote adaptability • Conduct cyclical small tests of change	Screening approach: couple-based reporting maximises clinical utility and minimises program complexity, saving laboratory and genetic counselling resources National variant review committee: enables access to specialist expertise across a wide range of rare conditions
Midpoint	Networks and Communication	• Promote network weaving • Organise clinician implementation team meetings • Build a coalition	Communication: between laboratory and clinical staff enables smooth operationalisation
Future	Cost	• Access new funding • Alter incentive/ allowance structures • Make billing easier	Funding: to include the cost of testing and quality pre- and post- test genetic counselling supports
	Adaptability	• Promote adaptability • Tailor strategies • Capture and share local knowledge	Access: adaptability of program considered from the outset Gene list iteration: laboratories need a mechanism to evolve the gene list over time
	Cosmopolitanism	• Capture and share local knowledge • Develop academic partnerships • Promote network weaving	Governance: authorities involved in the implementation of programs build on previous research and expertise

*CFIR* Consolidated Framework for Implementation Research, *ERIC* Expert Recommendations for Implementing Change.

One priority throughout, was **engaging** non-genetic HCPs, whose involvement in offering testing is imperative to scaling and sustaining population RGCS^4^. Previous research reports that the perceived **complexity** and intricacies of RGCS may lower HCP engagement^23,24^. In the Mackenzie’s Mission study, **complexity** was minimised through the **design quality and packaging** of the program which utilised technology to develop a streamlined model of care. HCPs considered this approach **compatible** with their current workflows and systems, which improved program **engagement**. Consistent with other research^25–27^, RGCS awareness was a contextual predictor of HCP engagement and created different **implementation climates** across study locations. Jurisdictions where HCPs had less prior experience with RGCS required a more hands-on approach from the study genetic counsellors early on. Understanding contextual factors such as, prior experience of an innovation crucial to ‘scaling up and out’ of programs^28^ and ensuring consistent uptake by HCPs^29^.

Another prominent priority in population RGCS is developing **consumer resources** to facilitate informed decision-making. Our findings triangulate with previous research^18^ that the **compatibility** of resources is important to **engagement** and community confidence in the program. The development, validation, and implementation of **consumer resources** was enabled through **access to knowledge** by housing the program within established organisations and drawing upon evidence of previous programs and pilot studies. Generating and **executing well-designed and packaged** information was considered **resource and time intensive**, especially when it was necessary to retrospectively **adapt** to different **consumer needs**. Implementation research recognises that the **adaptability** of program components is ideally recognised from the outset by understanding components by their *function* rather than their *form*^30,31^. Advanced planning can promote flexibility, delivering programs that are responsive to different environments while meeting the overall aims of RGCS screening. For example, translating resources into all languages reflective of Australia’s diverse population is unlikely to be the only avenue required to maximise **compatibility** and **engagement**^32^.

Although present across all implementation phases, having **resources available** was a priority for sustainability. Owing to the **complexities** of RGCS as an intervention^33–35^ laboratory and clinical workloads in some areas were greater than expected, revealing the reality that implementation in healthcare is not always straightforward, with differences between ‘*work as imagined*’ and ‘*work as done*’^36^. Maximising the **relative advantage** of a reproductive couple-based approach reflects previous research^37^ and reducing ambiguous results^38,39^ is likely key to sustainably addressing the reciprocal relationship between program complexity and the demand on laboratory and genetic workforces.

RGCS programs require a range of health care professionals and scientists to actively collaborate. **Networks and communication** were emphasised by participants as crucial to facilitating teamwork and collaboration. In addition, a more nuanced picture of teamwork in clinical genomics has been observed^40^. For example, how teamwork enables **access** to **expertise and knowledge**, through the reported success of the weekly variant review committee meeting and the benefits of a close working relationship between genetic counsellors and laboratory staff.

The limitation of conducting this research within a nationally funded program may hinder generalisability of findings to other less well-resourced implementation efforts. In addition, we recognise that variation in health system structures can affect how factors influence implementation outcomes. Nevertheless, by applying a widely adopted framework, our aim was to utilise a methodology that could be applied in other contexts, enhancing understanding. Although it is optimal to incorporate frameworks from the outset, here we retrospectively used the CFIR, as our intention was to identify shifts in priorities and factors influencing implementation rather than prospectively guide implementation. We acknowledge that being researchers situated within the project may have introduced bias, however, we consider the members of the CFIR team to be ‘accepted outsiders’ and that because of that position the team was able to collect longitudinal data and glean insights that may have otherwise been overlooked. Finally, we did not include the direct consumer experience, however consumer advocacy was captured through the Education and Engagement committee.

Our approach to integrating two datasets with overlapping sample participants within one implementation framework has captured both collective and short-term reflections and gained a longer-term and individual viewpoint. In doing so, this study provides a nuanced understanding of factors that promote or impede implementation of population RGCS. Of particular value are the insights for implementers looking to scale up and routinise RGCS.

## Materials and methods

### Study design

**Fig. 1:**
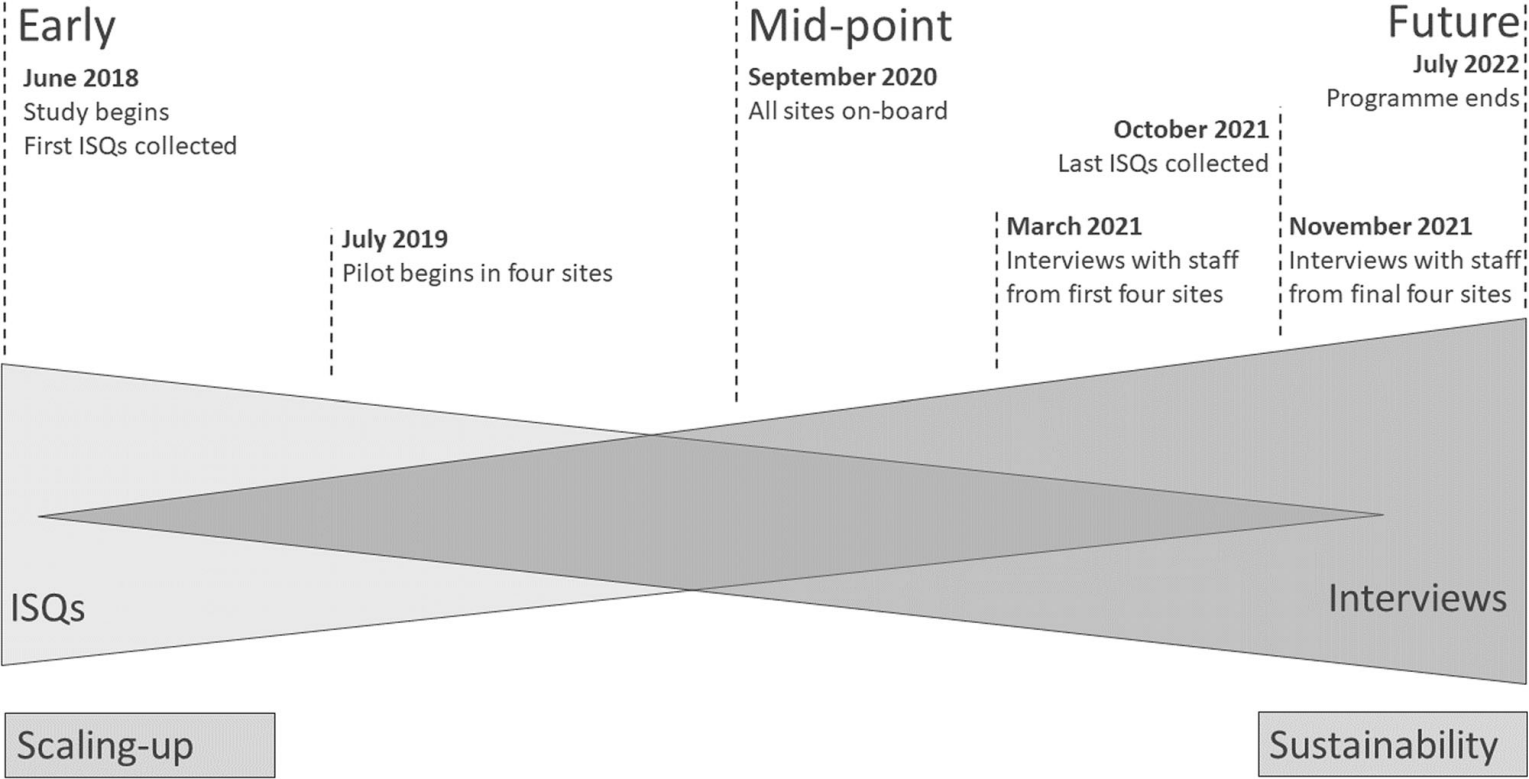
Conceptualisation of the two data collection methods (Implementation Science Questions (ISQs) and Interviews) and the relevance of data to the implementation period. The two triangles in this figure demonstrates the methods used to collect data and the expected content of each dataset with reference to the project timeline.

This study was part of a larger body of implementation research undertaken in the context of Mackenzie’s Mission. Here, we used a multi-method qualitative phenomenological approach^41^, with overlapping sample participants, integrating document analysis with semi-structured interviews. We employed a unique approach to collecting project data where members from 10 Mackenzie’s Mission committees were asked at the conclusion of regular meetings (mostly held monthly) to reflect on four questions, which we labelled ‘Implementation Science Questions’ (ISQs). This longitudinal qualitative approach provides a way of studying transitional and developmental work overtime^42^, and is especially useful in complex health service research^43^. We drew on a constructionist perspective to document analysis, acknowledging that the content of the ISQs is shaped by the context of their use and setting and interpret them through integration with the interview data. Figure 1 represents the two methods along the programme timeline and shows conceptually how they were integrated during analysis. The ISQs tracked the early to middle implementation phases, and interviews were used to gather reflections on how the Mackenzie’s Mission program functioned and perspectives on the future sustainability of RGCS programs. The study was approved by the Royal Children’s Hospital Melbourne, Human Ethics Committee (HREC/53433/RCHM-2019). Interview participants provided informed verbal consent prior to the interview commencing.

### Participants

ISQs were collected for 10 Mackenzie’s Mission committees (National Steering, Gene Selection, Laboratory, Variant Review, Education and Engagement, Recruitment, Clinical, Research Committee and Psychosocial and Epidemiology Subcommittee, National Operational, and one State Team). Committee members included representatives from all Australian jurisdictions, relevant peak professional bodies and professions involved in the delivery of RGCS programs. Committee membership remained consistent throughout the programme with high attendance rates. The only committee that had fluctuations in attendance was the weekly variant review committee and was dependant on the gene(s) that were being reviewed, on average there were 22 attendees but up to as many as 39 on two occasions. The Operational Team meeting expanded in line with the roll out across State and Territory jurisdictions. Supplementary Table 1 provides an overview of the committees, their purpose, membership, number of members, and meeting frequency.

For the interviews, individuals who had a joint role in the research component and involvement in the operation or service provision of Mackenzie’s Mission were invited via purposive sampling to ensure a national perspective and all eight study sites (State and Territory jurisdictions) were captured. This included staff directly employed through the study and clinical and laboratory staff engaged through participating services. Participants were invited via email, with one follow-up prompt and interviews scheduled at participants’ convenience.

### Data collection

The ISQs and interviews were run in parallel and did not inform each other. ISQs were the final standing agenda item and asked members to reflect on deliberately brief prompts to reduce barriers to completion (“what has gone well/not so well?”, “what has surprised you?” or “what have you learnt?”). The committee coordinator was also asked to comment on “what has changed?”. Following refinement after 1 year, “what has gone well/not so well?” was considered redundant and no longer asked. Responses were collected over 3 years from study initiation until towards the study end to allow for analysis (June 2018 – October 2021) and catalogued as an anonymous group reflection.

Interviews were guided by a semi-structured interview schedule (Supplementary Table 2) and were conducted reflexively so that the questions were appropriate to participants’ role and area of expertise. The interview guide was structured around the timepoints of interest (early, midpoint and future) and was revised partway through with no modifications made. Following verbal consent, interviews began by having participants describe their clinical area, involvement in Mackenzie’s Mission and prior experience of working in RGCS. Next, participants were asked to reflect on their experience starting off (early) “was there anything that would have made initiating Mackenzie’s Mission in your workplace easier?”. Once the program was underway (midpoint) “Now that Mackenzie’s Mission is underway, is there anything you find challenging about delivering the programme” and “have there been any unexpected consequences (positive or negative) from Mackenzie’s Mission?” Finally, participants were asked to think about the future and how they “felt genetic carrier screening should be provided?” and “what can facilitate this or what are the biggest barriers to implementing a national RGCS program?” Interviews were conducted between March and November 2021 by members of the research team with expertise in implementation science and qualitative research (ZF, SB, JL, and TT) who mostly had a prior professional relationship with participants. Interviews ran for 33 min on average (range 23–54) and were conducted via video conference, recorded, de-identified and transcribed verbatim by the research team.

### Data analysis

The two datasets were analysed using deductive content analysis^44^. A coding guide derived from the CFIR domains and constructs was developed with context specific definitions (Supplementary Table 3). Interview transcripts and ISQs were examined to identify factors influencing implementation within three distinct stages (early, midpoint, future). Following familiarisation with datasets, interviews were coded first, assigning sections of the transcript that aligned to the time period of enquiry to the CFIR code they best reflected. Four transcripts coded independently by two researchers (ZF and SB) and discrepancies discussed and resolved, and minor revisions to the coding guide made. One researcher (ZF) completed coding with regular discussions (SB) and began coding the ISQs with 10% undertaken independently (SB). The same approach to analysis was taken where each response to the ISQ prompt was assigned a CFIR construct and time period based on meeting date. Once analysed, regular group discussions (ZF, SB, and JL) were held to review challenging coding issues and an iterative process to identify relationships within the two datasets. Data were managed in NVivo 12 and Microsoft Excel.

### Reporting summary

Further information on research design is available in the Nature Research Reporting Summary linked to this article.

## Supplementary information

Reporting Summary

Supplementary Tables 1–4

## Data Availability

The datasets generated during and/or analysed during the current study can be made available from the corresponding author on reasonable request.
