# Supplementary material for: Scaling-up and future sustainability of a national reproductive genetic carrier screening program
Source: NPJ Genom Med. 2023 Jul 31;8:18. doi: 10.1038/s41525-023-00357-w (PMC10390466; doi:10.1038/s41525-023-00357-w)
Supplement: Supplementary file 1 — Reporting Summary [file 41525_2023_357_MOESM1_ESM.pdf]

nature portfolio

Corresponding author(s): Dr Stephanie Best
Last updated by author(s): Mar 31, 2023

Reporting Summary

Nature Portfolio wishes to improve the reproducibility of the work that we publish. This form provides structure for consistency and transparency in reporting. For further information on Nature Portfolio policies, see our Editorial Policies and the Editorial Policy Checklist.

Statistics

For all statistical analyses, confirm that the following items are present in the figure legend, table legend, main text, or Methods section.

n/a	Confirmed	
☒	☐	The exact sample size (*n*) for each experimental group/condition, given as a discrete number and unit of measurement
☒	☐	A statement on whether measurements were taken from distinct samples or whether the same sample was measured repeatedly
☒	☐	The statistical test(s) used AND whether they are one- or two-sided<br>*Only common tests should be described solely by name; describe more complex techniques in the Methods section.*
☒	☐	A description of all covariates tested
☒	☐	A description of any assumptions or corrections, such as tests of normality and adjustment for multiple comparisons
☒	☐	A full description of the statistical parameters including central tendency (e.g. means) or other basic estimates (e.g. regression coefficient) AND variation (e.g. standard deviation) or associated estimates of uncertainty (e.g. confidence intervals)
☒	☐	For null hypothesis testing, the test statistic (e.g. *F*, *t*, *r*) with confidence intervals, effect sizes, degrees of freedom and *P* value noted<br>*Give P values as exact values whenever suitable.*
☒	☐	For Bayesian analysis, information on the choice of priors and Markov chain Monte Carlo settings
☒	☐	For hierarchical and complex designs, identification of the appropriate level for tests and full reporting of outcomes
☒	☐	Estimates of effect sizes (e.g. Cohen's *d*, Pearson's *r*), indicating how they were calculated

Our web collection on statistics for biologists contains articles on many of the points above.

Software and code

Policy information about availability of computer code

Data collection	no software used
Data analysis	no software used

For manuscripts utilizing custom algorithms or software that are central to the research but not yet described in published literature, software must be made available to editors and reviewers. We strongly encourage code deposition in a community repository (e.g. GitHub). See the Nature Portfolio guidelines for submitting code & software for further information.

Data

Policy information about availability of data

All manuscripts must include a data availability statement. This statement should provide the following information, where applicable:

- Accession codes, unique identifiers, or web links for publicly available datasets
- A description of any restrictions on data availability
- For clinical datasets or third party data, please ensure that the statement adheres to our policy

The datasets generated during and/or analysed during the current study can be made available from the corresponding author on reasonable request.

Human research participants

Policy information about studies involving human research participants and Sex and Gender in Research.

Reporting on sex and gender	This information was not collected or reported in the study and is not relevant to the study aim
Population characteristics	See above
Recruitment	For the interviews, individuals who had a joint role in the research component and involvement in the operation or service provision of carrier screening through the study were invited via purposive sampling to ensure a national perspective and all eight study sites (State and Territory jurisdictions) were captured. This included staff directly employed through the study and clinical and laboratory staff engaged through participating services. Participants were invited via email, with one follow-up prompt. Selection bias was minimised by inviting all eligible participants.
Ethics oversight	The study was approved by the Royal Children's Hospital Melbourne, Human Ethics Committee (HREC/53433/RCHM-2019). Interview participants provided informed verbal consent prior to the interview commencing.

Note that full information on the approval of the study protocol must also be provided in the manuscript.

Field-specific reporting

Please select the one below that is the best fit for your research. If you are not sure, read the appropriate sections before making your selection.

☐ Life sciences ☒ Behavioural & social sciences ☐ Ecological, evolutionary & environmental sciences

For a reference copy of the document with all sections, see nature.com/documents/nr-reporting-summary-flat.pdf

Behavioural & social sciences study design

All studies must disclose on these points even when the disclosure is negative.

Study description	Multi-method qualitative phenomenological approach
Research sample	Implementation Science Questions (ISQs) were collected for 10 Mackenzie's Mission committees (National Steering, Gene Selection, Laboratory, Variant Review, Education & Engagement, Recruitment, Clinical, Research Committee and Psychosocial & Epidemiology Subcommittee, National Operational, and one State Team). Committee members included representatives from all Australian jurisdictions, relevant peak professional bodies and professions involved in the delivery of carrier screening programs. Committee membership remained consistent throughout the programme with high attendance rates. The only committee that had fluctuations in attendance was the weekly variant review committee and was dependant on the gene(s) that were being reviewed, on average there were 22 attendees but up to as many as 39 on two occasions. The Operational Team meeting expanded in line with the roll out across State and Territory jurisdictions.<br>For the interviews, individuals who had a joint role in the research component and involvement in the operation or service provision of the study were invited via purposive sampling to ensure a national perspective and all eight study sites (State and Territory jurisdictions) were captured. This included staff directly employed through the study and clinical and laboratory staff engaged through participating services.
Sampling strategy	We interviewed all eligible participants who consented to an interview, thus our sample size was predetermined and data saturation not applicable
Data collection	Qualitative study: blinding not applicable<br><br>Two data collection methods were used.<br>ISQs were the final standing agenda item and asked members to reflect on deliberately brief prompts to reduce barriers to completion ("what has gone well/not so well?", "what has surprised you?" or "what have you learnt?"). The committee coordinator was also asked to comment on "what has changed?". Following refinement after one year, "what has gone well/not so well?" was considered redundant and no longer asked. Responses were catalogued as an anonymous group reflection.<br>Interviews were guided by a semi-structured interview schedule and were conducted reflexively so that the questions were appropriate to participants' role and area of expertise. The interview guide was structured around the timepoints of interest (early, midpoint and future) and was revised partway through with no modifications made. Following verbal consent, interviews began by having participants describe their clinical area, involvement in Mackenzie's Mission and prior experience of working in RGCS. Next, participants were asked to reflect on their experience starting off (early) "was there anything that would have made initiating Mackenzie's Mission in your workplace easier?". Once the program was underway (midpoint) "Now that Mackenzie's Mission is underway, is there anything you find challenging about delivering the programme" and "have there been any unexpected consequences (positive or negative) from Mackenzie's Mission?" Finally, participants were asked to think about the future and how they "felt genetic carrier screening should be provided?" and "what can facilitate this or what are the biggest barriers to implementing a national RGCS program?" Interviews were conducted between March and November 2021 by members of the research team with expertise in implementation science and qualitative research (ZF, SB, JL, and TT) who mostly had a prior

	professional relationship with participants. Interviews ran for 33 minutes on average (range 23 –54) and were conducted via video conference, recorded on a digital recorded, de-identified and transcribed verbatim by the research team.
Timing	Implementation Science Questions were collected for meeting held between June 2018 – October 2021<br>Interviews were conducted between March and November 2021
Data exclusions	No data were excluded from analysis
Non-participation	4 participants passively declined participating in an interview through non-response to the invite.
Randomization	No randomisation was used

Reporting for specific materials, systems and methods

We require information from authors about some types of materials, experimental systems and methods used in many studies. Here, indicate whether each material, system or method listed is relevant to your study. If you are not sure if a list item applies to your research, read the appropriate section before selecting a response.

Materials & experimental systems

n/a	Involved in the study	
☒	☐	Antibodies
☒	☐	Eukaryotic cell lines
☒	☐	Palaeontology and archaeology
☒	☐	Animals and other organisms
☒	☐	Clinical data
☒	☐	Dual use research of concern

Methods

n/a	Involved in the study	
☒	☐	ChIP-seq
☒	☐	Flow cytometry
☒	☐	MRI-based neuroimaging
