# Supplementary material for: Scaling-up and future sustainability of a national reproductive genetic carrier screening program
Source: NPJ Genom Med. 2023 Jul 31;8:18. doi: 10.1038/s41525-023-00357-w (PMC10390466; doi:10.1038/s41525-023-00357-w)
Supplement: Supplementary file 2 — Supplementary Tables 1–4 [file 41525_2023_357_MOESM2_ESM.pdf]

Supplementary Table 1: Overview of the committees involved in Mackenzie's Mission.

**National Steering Committee: quarterly**<br>*Responsible for providing strategic guidance and overall management of the study.*	**Six members**<br>The study co-leads<br>The lead and manager of Australian Genomics<br>The mother of Mackenzie, who the study is named after
**Laboratory Committee: monthly**<br>*Responsible for the development and delivery of the genetic testing required for the program.*	**Seventeen members**<br>Senior and Junior laboratory scientists<br>Genetic pathologists<br>Medical scientists and technical assistants at the three participating laboratories<br>Genetic counsellors/state coordinators
**Recruitment Committee: every 3 weeks**<br>*Works with the study's Operational Team to develop strategies and plans for the recruitment of healthcare providers and participants into the study.*	**Nine members**<br>Healthcare providers with experience in reproductive healthcare and screening
**Clinical Committee: monthly**<br>*Works with the study's genetic counsellors to develop participant consent forms, monitor recruitment of healthcare providers and participants, and oversee the clinical management of participants who have genetic carrier screening through the study.*	**Nine members**<br>Leads from each state and territory<br>Genetic counsellors
**Research Committee: monthly**<br>*Oversees the Mackenzie's Mission research program, which includes four key streams: Psychosocial and Epidemiology, Implementation Science, Bioethics and Health Economics.*	**Nine members**<br>Senior researchers and leads from each of the four research streams
**Education & Engagement Committee: fortnightly for Year 1 and as required thereafter**<br>*Developing study education materials and producing information about Mackenzie's Mission to inform the general public and developing content for the study website.*	**Thirteen members**<br>Medical educators<br>General practitioners<br>Clinical geneticist<br>Genetic counsellors<br>A community representative
**Gene selection Committee: fortnightly for Year 1 and as required thereafter**<br>*Carries out a comprehensive evaluation of genes relevant to reproductive genetic carrier screening and developed the list of genes and genetic conditions to be screened through the study.*	**Ten members**<br>Clinical geneticists<br>A genetic pathologist<br>A medical scientist<br>A genetic counsellor<br>An obstetrician<br>An ethicist<br>A parent of a child with a genetic condition
**Variant Review Committee: weekly**<br>*Reviewing variants which arise from the study that are difficult to interpret.*	**Twenty-two members (average)**<br>Clinical geneticists<br>Genetic pathologists<br>Medical scientists<br>Genetic counsellors
**Psychosocial & Epidemiology subcommittee: monthly**<br>*Develop research program and ensures research milestones are met.*	**Six members**<br>Project leads<br>Researchers
**National operational team: fortnightly**<br>*Ensure that activity progresses consistently and that study milestones are met.*	**Sixteen members**<br>Project coordinators and administrators<br>State genetic counsellors<br>Psychosocial and implementation science research teams

**State operational team: monthly**<br>*Ensures communication between clinical and laboratory team.*	**Nineteen members**<br>State project lead<br>Project coordinator and administrator<br>State laboratory team<br>State genetic counsellors

Supplementary Table 2: Interview Schedule used to guide semi-structured interviews with members of the study team.

Area of interest	Sample questions
**Context**	What area to do you work in and what does this involve?
**Starting off Mackenzie's Mission**	Before Mackenzie's Mission (Mackenzie's Mission) did you have any previous experience of working in reproductive carrier screening?<br>Starting off was there anything that would have made initiating Mackenzie's Mission in your workplace easier?
**Once underway**	Now Mackenzie's Mission is underway, is there anything you find challenging about delivering the programme?<br>Or is helpful when thinking about facilitating a reproductive carrier screening programme?<br>How has Mackenzie's Mission impacted on the rest of your daily work?<br>Have there been any unexpected consequences (positive or negative) of offering Mackenzie's Mission in the overall productivity/procedures/operation of the clinic/lab?
**Looking to the future**	How do you feel reproductive carrier screening should be provided in the future?<br>What will facilitate this?<br>From your experience to date, what do you think will be the biggest barriers to implementation of a nationwide reproductive carrier screening programme?<br>What advice would you give to new lab/clinic about implementing a reproductive carrier screening programme?

Supplementary Table 3: Consolidated Framework for Implementation Science coding guide with definitions and context specific definitions.

CFIR constructs by domain	**Definition** Damschroder L et al., (2009)[1]	**Definition in RGCS context**
I. Characteristics of RGCS: *the intrinsic nature of RGCS or RGCS program componentry*		
**A. Intervention Source**	Perception of key stakeholders about whether the intervention is externally or internally developed.	The perceived qualities (including expertise, and credentials) of the individuals involved in developing the RGCS program.
**B. Evidence Strength & Quality**	Stakeholders' perceptions of the quality and validity of evidence supporting the belief that the intervention will have desired outcomes.	Perceived quality and validity of the evidence supporting the RGCS program and that the program will have the desired outcomes
**C. Relative Advantage**	Stakeholders' perception of the advantage of implementing the intervention versus an alternative solution.	Perceived advantage of implementing an accessible, couple based, expanded national RGCS vs other options for implementing RGCS programs (e.g., payment, smaller screening panels, individual testing).
**D. Adaptability**	The degree to which an intervention can be adapted, tailored, refined, or reinvented to meet local needs. Adaptability relies on a definition of the 'core components' (the essential and indispensable elements of the intervention itself) versus the 'adaptable periphery' (adaptable elements, structures, and systems related to the intervention and organization into which it is being implemented) of the intervention	Perceived degree to which components of a RGCS program can be adapted, tailored, refined, or reinvented to meet geographic, clinical, lab, HCPs, operational or patient needs.
**E. Trialability**	The ability to test the intervention on a small scale in the organization, and to be able to reverse course (undo implementation) if warranted.	Perceived ability to and evidence of testing components of a RGCS program on a small scale, and to be able to reverse course (undo implementation) if warranted.
**F. Complexity**	Perceived difficulty of implementation, reflected by duration, scope, radicalness, disruptiveness, centrality, and intricacy and number of steps required to implement.	Perceived difficulty of implementation, reflected by duration, scope, radicalness, disruptiveness, centrality, and intricacy and number of steps required to implement the RGCS program.
**G. Design Quality & Packaging**	Perceived excellence in how the intervention is bundled, presented, and assembled	Perceived excellence in how the components of the RGCS program are bundled, presented, and assembled.
**H. Cost**	Costs of the intervention and costs associated with implementing the intervention including investment, supply, and opportunity costs	Perceived costs of the RGCS program and associated costs with implementing a RGCS program.
II. Outer setting: *organisations and stakeholders who are indirectly involved in a RGCS program*		
**A. Patient Needs & Resources**	The extent to which patient needs, as well as barriers and facilitators to meet those needs, are accurately known and prioritized by the organization.	The extent to which patient needs, as well as barriers and facilitators to meet those needs, are accurately known and prioritized by the involved organisations/teams in implementing the RGCS program.

**B. Cosmopolitanism**	The degree to which an organization is networked with other external organizations.	The degree and nature to which the involved organisations/teams are networked with other external organizations and relevant bodies
**C. Peer Pressure**	Mimetic or competitive pressure to implement an intervention; typically because most or other key peer or competing organizations have already implemented or are in a bid for a competitive edge.	Mimetic or competitive pressure to implement a national RGCS, mindful of existing and potential pressures to implement RGCS in different contexts i.e., the private and non-private sector
**D. External policies, Regulations & factors**	A broad construct that includes external strategies to spread interventions, including policy and regulations (governmental or other central entity), external mandates, recommendations and guidelines, pay-for-performance, collaboratives, and public or benchmark reporting.	A broad construct that includes external strategies to spread interventions, including policy and regulations and factors outside of the control of the study team that impact the implementation of a RGCS program including media, competing public health priorities, economic climate etc.
III. Inner setting: *of a RGCS program. Study team and stakeholders directly involved i.e., health care professionals*		
**A. Structural Characteristics**	The social architecture, age, maturity, and size of an organization.	The social architecture, governance structure, workforce recruitment, and experience of the teams involved in implementing a RGCS program.
**B. Networks & Communications**	The nature and quality of webs of social networks and the nature and quality of formal and informal communications within an organization.	The nature and quality of formal and informal pathways, and communications between teams involved in implementing a RGCS program.
**C. Culture**	Norms, values, and basic assumptions of a given organization.	The collective norms, values, and basic assumptions of teams involved in implementing a RGCS program.
**D. Implementation Climate**	The absorptive capacity for change, shared receptivity of involved individuals to an intervention, and the extent to which use of that intervention will be rewarded, supported, and expected within their organization.	Any reference to a perceived absorptive capacity for change, receptivity to implementing a RGCS program, and the extent to which RGCS will be supported and expected within the teams directly involved and the wider community (including general public, healthcare professionals and genetics community)
D1. Tension for Change	The degree to which stakeholders perceive the current situation as intolerable or needing change.	The degree to which participants perceive the current availability of RGCS as intolerable or needing change.
D2. Compatibility	The degree of tangible fit between meaning and values attached to the intervention by involved individuals, how those align with individuals' own norms, values, and perceived risks and needs, and how the intervention fits with existing workflows and systems.	The degree to implementing a RGCS program fits with existing workflows and systems.
D3. Relative Priority	Individuals' shared perception of the importance of the implementation within the organization.	Teams' shared perception of the importance of implementing a RGCS program
D4. Organizational Incentives & Rewards	Extrinsic incentives such as goal-sharing awards, performance reviews, promotions, and raises in salary, and less tangible incentives such as increased stature or respect.	Extrinsic incentives such as goal-sharing awards, performance reviews, promotions, and raises in salary, and less tangible incentives such as increased stature or respect.

D5. Goals and Feedback	The degree to which goals are clearly communicated, acted upon, and fed back to staff, and alignment of that feedback with goals.	The degree to RGCS goals are clearly communicated, acted upon, and fed back to staff, and alignment of that feedback with goals.
D6. Learning Climate	A climate in which: a) leaders express their own fallibility and need for team members' assistance and input; b) team members feel that they are essential, valued, and knowledgeable partners in the change process; c) individuals feel psychologically safe to try new methods; and d) there is sufficient time and space for reflective thinking and evaluation.	The climate around a RGCS program that allows: a) leaders to express leaders express their own fallibility and need for team members' assistance and input; b) team members feel that they are essential, valued, and knowledgeable partners in the change process; c) individuals feel psychologically safe to try new methods; and d) there is sufficient time and space for reflective thinking and evaluation.
**E. Readiness for Implementation**	Tangible and immediate indicators of organizational commitment to its decision to implement an intervention.	Tangible and immediate indicators of involved individuals, or the broader community's commitment to implementing a RGCS program.
E1. Leadership Engagement	Commitment, involvement, and accountability of leaders and managers with the implementation.	Commitment, involvement, and accountability of leaders and managers with the implementation of a RGCS program.
E2. Available Resources	The level of resources dedicated for implementation and on-going operations, including money, training, education, physical space, and time.	The resources dedicated for implementation of a RGCS program and on-going operations, including money, training, education, physical space, and time.
E3. Access to Knowledge & Information	Ease of access to digestible information and knowledge about the intervention and how to incorporate it into work tasks.	Ease of access to information and knowledge about RGCS and how to incorporate it into work tasks.
IV. Characteristics of individuals involved in running a RGCS program		
**A. Knowledge & Beliefs about the Intervention**	Individuals' attitudes toward and value placed on the intervention as well as familiarity with facts, truths, and principles related to the intervention.	Individuals' attitudes toward and value placed on a RGCS program as well as familiarity with facts, truths, and principles related to RGCS.
**B. Self-efficacy**	Individual belief in their own capabilities to execute courses of action to achieve implementation goals.	Individuals or teams' collective belief in their own capabilities to execute courses of action to implement a RGCS program
**C. Individual Stage of Change**	Characterization of the phase an individual is in, as he or she progresses toward skilled, enthusiastic, and sustained use of the intervention.	The phase an individual is perceived to be in, as they progress toward skilled, enthusiastic, and sustained delivery of RGCS.
**D. Individual Identification with Organization**	A broad construct related to how individuals perceive the organization, and their relationship and degree of commitment with that organization.	How individuals perceive the involved organization/teams and their relationship and degree of commitment with the organization/team involved in a national RGCS program.
**E. Other Personal Attributes**	A broad construct to include other personal traits such as tolerance of ambiguity, intellectual ability, motivation, values, competence, capacity, and learning style.	Personal traits such as tolerance of ambiguity, intellectual ability, motivation, values, competence, capacity, and learning style.
V. Process of establishing and running a RGCS program		

**A. Planning**	The degree to which a scheme or method of behavior and tasks for implementing an intervention are developed in advance, and the quality of those schemes or methods.	The degree of planning prior and during the implementation of a national RGCS program, and the perceived quality of the planning.
**B. Engaging**	Attracting and involving appropriate individuals in the implementation and use of the intervention through a combined strategy of social marketing, education, role modelling, training, and other similar activities.	The process of attracting and involving relevant stakeholders (advocacy groups, Health care providers, and consumers) in the implementation and use of RGCS.
B1. Opinion Leaders	Individuals in an organization who have formal or informal influence on the attitudes and beliefs of their colleagues with respect to implementing the intervention.	Individuals from involved organizations/teams who are influential (formally or informally) on the attitudes and beliefs of others involved in a RGCS program.
B2. Formally Appointed Internal Implementation Leaders	Individuals from within the organization who have been formally appointed with responsibility for implementing an intervention as coordinator, project manager, team leader, or other similar role.	Individuals from within the study team who have been formally appointed responsibility to implement a RGCS program
B3. Champions	"Individuals who dedicate themselves to supporting, marketing, and 'driving through' an [implementation]" [101] (p. 182), overcoming indifference or resistance that the intervention may provoke in an organization.	Individuals outside of leadership roles who dedicate themselves to implementing a RGCS program.
B4. External Change Agents	Individuals who are affiliated with an outside entity who formally influence or facilitate intervention decisions in a desirable direction.	Individuals from an outside entity who have a formal, influence or enables implementing a RGCS program.
**C. Executing**	Carrying out or accomplishing the implementation according to plan.	Carrying out or accomplishing the implementation of the RGCS program according to plan.
**D. Reflecting & Evaluating**	Quantitative and qualitative feedback about the progress and quality of implementation accompanied with regular personal and team debriefing about progress and experience.	Formal and informal processes of reflecting and evaluating that influence the progress the quality of the implementation or results in a change in action being taken.

[1] Footnote: Damschroder, L. J. et al. Fostering implementation of health services research findings into practice: a consolidated framework for advancing implementation science. Implement. Sci. 4, 50 (2009).

Abbreviations: CFIR (Consolidated Framework for Implementation Research); RGCS (Reproductive Genetic Carrier Screening); HCPs (Health Care Professionals)

Supplementary Table 4: Exemplar ISQs and interview quotes (***Bold and italic text denotes interviews*** and plain text ISQs)

CFIR Construct	Early	Middle	Future
**CFIR Domain** Intervention Characteristics: *the intrinsic nature of RGCS or specific program components*			
Intervention Source	***Working with the brightest and the best in Australia was huge…just the depth and the breadth of knowledge that everyone at the table has is amazing. So, I thought it was set-up even better than I could have ever imagined.*** **(Coordination 06)**		***It was almost like the decision [at the government level] about the model to delivering this [as a funded test rather a national program] was almost decided before the program even began and so in a way it's a bit of shame that that decision was made and I don't know how informed that decision was and whether the person who made that decision understands the complexities of genetics.*** **(Coordination 03)**
Evidence Strength and Quality			***There's quite a bit of work that needs to be done to translate this technology to be appropriate in the screening context because I think there's risk that we are doing harm to people by reporting variants that probably don't really apply.*** **(Coordination 03)**
Relative Advantage	Positive feedback on couple-based screening approach. (Operational team)	Comments from HCPs on preference for a national screening program rather than an item number. (Research Committee)	***It's just that missed opportunity [with couple-based] for cascade testing through the family. BUT resources are finite, and we would need about a thousand more genetic counsellors if you wanted to give individual risk results, so I understand that's not possible.*** **(Coordination 06)**<br><br>***Definitely with couple results rather than individual results, that was something that was definitely a huge advantage in terms of workload… So, I think, any future program should be based in the same way where you report couple results.*** **(Lead 04)**<br><br>***Mackenzie's Mission has worked cost-effectively because it's couples and you can report on the couples*** **(Lead 02)**

			In an ideal world it would be a national program. Because a national program would be able to deliver consistency in terms of how it is delivered, what we are screening for, and address some of these equity of access issues. In reality, it is going to be a [public] funded test, which means you are purely funding the test and not the service that sits around it. The service that sits around it, is really probably 80% of the whole thing. **(Coordination 03)**<br><br>***I think GPs are the best ways to do it, by far because they are the place where people go, and they probably go there for health care before they are pregnant. And I think ideally you would want this before you are pregnant.*** **(Lead 03)**<br><br>***The everyday person, with no family history…I don't think we [genetic services] have capacity to see them and consent them or coordinate that testing for them. So, I think the GPs are ideally placed for those people, but I understand they probably don't have the capacity either and some of them probably don't have the want to do it either.*** **(Coordination 05)**
Adaptability	As we go into accessibility phase, there are a lot of things to consider and a lot of changes we will need to make to adapt to different contexts. (Psychosocial & Epidemiology Committee)<br><br>Changes to recruitment processes to adapt to COVID-19, e.g., recruitment form, strategies. (Research Committee)	Material has been translated into Arabic. (Education & Engagement Committee)<br><br>COVID-19 has impacted the way we deliver education in some positive ways (flexibility, lower resource use, geographic spread, 'catch-up' sessions). (Education & Engagement Committee)	***We also take an outreach nurse…to share knowledge more broadly with the health staff there [in communities] So, directly building workforce capacity there but also directly to groups of people. So, it would be wonderful, and I could envision a situation, if we were appropriately resourced, to have a doctor and or nurse/midwife to go out and provide this [RGCS] education sessions in a more holistic way and support the primary health care approach in that way. And then again, this is another question, do we really need to go out there or could we do it by Telehealth?*** **(Snr Member 01)**

			We haven't even got into metro, rural and in particular Aboriginal community, again how do we tap into that is difficult questions. I think we are going to have to develop strong community programs, whether that is at school…or we have breast screen vans rolling into community and whether we do this with genetic carrier screening to create and awareness. **(Lead 05)**<br><br>***Another one of those barriers we need to look at is being able to get everyone access in terms of languages and translating the material.*** **(Coordination 06)**
Trialability	Decision Aid complete and ready for trialling. (Education & Engagement Committee)<br><br>Identified needs for delivery of HCP education. Potential to develop a tailored approach to trial and revise. (Operational team)	The difficulties of de-implementation. Once genes are on a screening panel, they are hard to take off. The importance of gene list version control. (Gene Selection Committee)<br><br>We made a few mistakes in gene selection - genes associated with severe conditions may not be suitable for carrier screening e.g., due to a lack of correlation between genotype and phenotype => importance of flexibility in the gene panel and the value of road-testing the panel thoroughly. (Variant Review Committee)	
Complexity	Preparing gene list is a huge and somewhat daunting task. (Executive Committee)	Panels in diagnostic context don't necessarily relate to panels created for a different context. (Gene Selection Committee)<br><br>A pragmatic reduction of panel size would not be simple - on what basis would we draw a line for excluding genes - complexity of analysis/counselling, residual risk, frequency. (Gene Selection Committee)	For a small increase in cost, labs can screen twice the number of genes. However, more is not always better - very rare genes do not align with screening principles of having robust evidence/information. (Laboratory Committee)<br><br>The laboratories have to make tricky decisions for borderline VUS/LP variants - variant classification is not easy, and is different in the screening to the diagnostic context - made even harder by the high-

		'Increased chance' couples with definitive results are easier to counsel. Results where the condition has the potential for high variability can be more difficult. (Operational team)<br><br>Where couples already have children, an increased-chance result often has implications for those children e.g., if condition is variable / has onset in later childhood. This is an element of expanded carrier screening that doesn't occur in cystic fibrosis/spinal muscular atrophy/Fragile X syndrome screening, where couples know early on if they have an affected child. (State team)<br><br>***A result that we gave and the gene involved we couldn't say whether the baby affected was going to be significantly affected in childhood or maybe even in utero or have a mild condition in adulthood and that was really tricky. I had kind of expected that if the genes were on the list that they would be really clear cut.* (Study GC 02)**<br><br>***People have done this testing after they've had a child or a few children beforehand so there's consequences for those children, potentially they're affected with this condition if it's a milder thing…and we haven't really thought about the time that it would take with the couples and how much of an impact on our time that would have because of the complexities that people bring*. (Study GC 01)**	throughput, fast TAT demands of screening. (Variant Review Committee)<br><br>***Turning this into a big program I think we need fewer genes initially and we need to just work out any way of automating it*. (Lead 01)**<br><br>***I feel it's going to be a complex thing trying to make the process, if it was available to all, simple yet comprehensive and that is going to be a big challenge going forward.* (Coordination 04)**<br><br>***If there's too much grey in a screening program firstly, it's hard to communicate the information people need to know up-front, so it's hard for them to make an adequate and informed decision about screening*. (Coordination 03)**<br><br>***It is letting go of the perfection that we would seek and having the best for the most. So, I think the implementation is going to be about, what level of information do people get, what level of interpretation can be provided and the proviso that are given.* (Lead 03)**
Design Quality & Packaging	Flow of couples' material is clear and thought-through. (Education & Engagement Committee)	***The whole process of recruitment is so streamlined and fast, that's actually a reduction in their [health care professionals'] time*. (Lead 04)**	***I think the front end is fantastic… I think we've got to minimise the Genetic Counsellor requirement at the front end by having fantastic resources for people to get the info, the bots and all those sorts of things to answer questions… there is more than***

	Positive that a large component was already online, and therefore appropriate for telehealth consultations. (Recruitment Committee)<br><br>***I think we could have taken more advantage of the expertise we had…it's actually the little things that make a big difference when implementing a program. It's the little details like – what do your test kits need to be like, and how are you going to mail them out, and what's your education going to be like…and how do you make it really simple or straightforward for your target population. Sometimes we think too much about the higher-level stuff and we don't give enough attention to the details.* (Coordination 03)**	***I think the study has been really cleverly designed to make it as easy as possible for recruiters…That's been a really important process…So once GPs actually understood it, it's quite simple and probably only added 5 or 10 mins onto their consult. That again helped with their willingness.* (Lead 05)**<br><br>***From the couples' perspective I think we have set-up a really nice, easy to follow program.* (Coordination 02)**<br><br>***The sample collection is going alright…the collection instructions are very good. I know that Mackenzie's Mission put a lot of time and thought into those collection instructions, and the images are really great.* (Laboratory 02)**<br><br>***How difficult it was to develop a postage kit and sort that all. But now it's running smoothly.* (Coordination 01)**	***7000 pregnancies a year in Australia that are conceived at 1 in 4 risk of a recessive or x-linked condition that can be picked up from this type of screening. That is a whole lot of work for Genetic Counsellors so that's where the energy should be maximised.* (Lead 01)**<br><br>***It's been a real blessing in hindsight, the way that the project was designed to be accidentally COVID proof. With everything happening online and with the postal kits. I think it's really kind of proved that model. And now with the way that healthcare has been probably influenced by COVID, I think it will be more a more and more acceptable to people to do something online and to not necessarily have face to face contact with anyone.* (Coordination 01)**
Cost	How costly it is in CTRL [an online research consent portal]. (Psychosocial & Epidemiology Committee)		***$20 million is going to be used up maybe testing 10,000 [couples] if we are lucky but probably not. And at the end of it we are all going to say we could of used more staffing…ok fine maybe the labs might have been resourced enough and some might not be…so where does that money come from?* (Coordination 02)**<br><br>***The more grey you have, the more resources you have to put in to sorting it out and the more expensive the program becomes as a whole. That's why when you're doing something at scale you have to keep it really simple.* (Coordination 03)**

CFIR Domain Outer Setting: *organisations and stakeholders who are indirectly involved in a RGCS program*

Patient Needs & Resources	Group recognised need to develop a protocol to follow-up with increased psychosocial risk couples (Psychosocial & Epidemiology Committee)<br><br>***In my experience, one of the barriers to carrier screening has been cost for the couples. So, it has been fantastic to be involved in this study which may see real positive outcomes in terms of equity for couples in this area*. (Coordination 04)**	The length of time it has taken Mackenzie's Mission to address accessibility of the project in terms of languages other than English, and in format for sight impaired individuals. (Engagement Committee)<br><br>The development of translated materials is becoming a priority. (Operational team)<br><br>Non-English speaking couple enrolment isn't practical using the current process. (Psychosocial & Epidemiology Committee)<br><br>The amount of time [it takes] to recruit a non-English speaking couple show the benefit of having translated materials. (Operational team Committee)<br><br>Surprised that with the [translated] Arabic portal available, participants would prefer to use the English portal with phone assistance. (Operational team)<br><br>Mackenzie's Mission [is] distinctive in that many other studies do not have the capacity to recruit non-English-speaking participants. (Operational team)<br><br>The experience with the donor couple using the portal shows why a specialised enrolment pathway is needed for these groups. (Operational team)<br><br>***I think if it's going to be rolled out nationally, and I don't know if we had different materials [but] they've got a really cool Aboriginal designed poster* (Lead 03)**	Enrolling participants through alternative pathways highlights the importance of genetic counsellors being involved in reproductive genetic carrier screening, we need more than just a lab number. (Operational team Committee)<br><br>***I wonder whether if there are people from a non-English speaking background who don't get offered Mackenzie's Mission because the doctor might think it's too hard or whatever, or they don't have the resources or time to sit down and actually enrol. So how to make it accessible, that is the big question.* (Coordination 04)**<br><br>***If we want the highest uptake possible it has to be free, it has to be subsidised. We know from our own experience with first trimester combined screening and now NIPT that whilst the uptake is good, it certainly is not 100% largely because there is a cost involved. So, it has to be publicly funded.* (Lead 05)**

Cosmopolitanism	The level of engagement in the Delphi group in designing the Decision Aid. (Psychosocial & Epidemiology Committee)<br><br>Dynamic with patient support groups – need to think about other support groups (Executive Committee)<br><br>Useful meeting and interactions at HGSA (Human Genetics Society of Australasia) conference. (Laboratory Committee)	New collaboration with European carrier screening consortium. (Executive Committee)<br><br>Symposium held with Dutch & Belgian carrier screening groups highlighted the large scale of Mackenzie's Mission and what a great opportunity this project is. Found these groups are encountering the same issues/considerations as we are. (Executive Committee)	
External Policies & Regulations			***I think that at the moment the big barriers are awareness of the health care professional and awareness of the community but once there is a either free or cheap test I think that will shift really quickly…And the guidelines currently say that it should be offered. It will get to the point where it will be considered negligent if they didn't offer it.*** **(Coordination 03)**
**CFIR Domain** Inner Setting: *of a RGCS program. Study team and stakeholders directly involved i.e., health care professionals*			
Structural Characteristics	Not enough coordination or admin capacity, but this is being addressed. (Executive Committee)<br><br>***I would have had more operational input. It's not about understanding it academically but actually understanding how do you roll this out as a program?…If we had also employed some more research assistants in that first year instead of genetic counsellors, then it would have been easier to develop a lot of our data collection tools and the portal.*** **(Coordination 03)**<br><br>Now that senior scientist [is] on board things are moving. (Laboratory Committee)	Need for administrative role to deal with incorrect samples and issues with test request forms. (Operational team)<br><br>***We have a dedicated team - all we do is Mackenzie's Mission so from that point of view there's no problem in terms of the integration with the rest of the Department .*** **(Laboratory 01)**<br><br>***We sort of outsourced the actual sequencing and paid for that separately, it's not within the Mackenzie's Mission team and there were issues with that that was a brand-new service. It just took time to get going. The lab didn't have a sufficient staff. So we actually had a lot of samples just***	

		waiting to be sequenced and that was a big bottleneck.* (Coordination 01)**<br><br>Would have been ideal to have more admin support from the very beginning so I guess the three counsellors for about a year we were doing all the admin…so it would have been good to have that set up from the beginning and just the foresight…to have thought about how we were going to deal with couple inquiries.* (Study GC 01)**	
Networks & Communications	Not [done] so well – General communication across project. (Executive Committee)<br><br>***It's hard to establish really good working relationships with people when you are not working in the same location as them on a daily basis, so having a more middle structure would have helped with that because there would have been people more able to be more hands on and create better lines of communication across the states too*. (Coordination 03)**<br><br>Communication can be improved about disseminating changes in committees to relevant parties. (Laboratory Committee)<br><br>A lot more collaboration between the labs now. (Executive Committee)<br><br>Data collection tools have been developed rapidly, good teamwork & communication. (Psychosocial & Epidemiology Committee)<br><br>[Did] Not [go] so well - sometimes difficult to disseminate drafts and encourage collaboration. (Education & Engagement Committee)	Collaboration between committees to coordinate and strengthen recruitment approach in a research-based way. (Research Committee)<br><br>The need for and the effect on the Operations Team when the lab doesn't run 100% smoothly. (Operational team)<br><br>***To have that forum [variant review committee]. It's definitely kept kind of all the states collaborating and talking to each other.* (Coordination 02)**<br><br>***Working quite closely with the lab is really important and to have those links because you need to be able to communicate information about family histories and the results and things.*<br>(Study GC 02)**<br><br>***We…meet once a fortnight and the lab is at that meeting. That has allowed for real collaboration and a lot of flow of information to happen. So even though we are not there, it feels like you have a fairly good idea of what's going on in the lab and where they are at*. (Coordination 05)**	***You can't keep doing an MDT [multi-disciplinary team] for every pregnant couple in Australia… As soon as there is any doubt it's a VUS (Variant of Unknown Significance), then there can't be someone emailing the world's expert on something to just see what they think.* (Lead 03)**

	Discussion functioning well, collective wisdom remarkable. (Variant Review Committee)	***I don't know how many GPs would regularly refer people to genetic services or even call them because they have got a question…this program fosters a good relationship with those who are offering the test to know they are supported by experts in the field, if needed*. (Coordination 02)**	
Implementation climate	Enthusiasm from HCPs who have been approached. (Recruitment Committee)<br><br>GP enthusiasm to recruit despite challenges. (Recruitment Committee)<br><br>Lack of understanding about genetic testing amongst GPs. (Education & Engagement Committee)<br><br>Supportiveness of the IAG [International Advisory Group]. (Executive Committee)<br><br>From conference – awareness of how much Mackenzie's Mission is in the interest of clinical and lab community across Australia. (Executive Committee)<br><br>***Everybody in the human genetics community and community in Australia wants this. They want carrier screening. Oh, there might be some…we approached [one health service] and they refused to do it…I think that is one of the biggest barriers to everyone in Australia accessing carrier screening.* (Lead 02)**<br><br>***Given how keen our GPs were, it didn't take much to sell to GPs and obstetricians around town. So a couple of lectures and calls to get a core group of people involved.* (Lead 05)**	Interesting to see how well recruitment has gone in Tasmania; this is likely due to minimal COVID impacts there. On the other hand, establishing recruitment in Northern Territory has been difficult; believe this is due to lack of awareness of RGCS and high turnover of HCPs. (State team)<br><br>We have supportive HCPs who strongly believe in the value of the study. (State team)<br><br>***Other states where screening isn't being done and isn't really thought about, they have struggled a lot to get couples*. (Lead 01)**	

	For me easy. I actually have not found it that hard at all [engaging HCPs]. But that might be a function of being [in a] small [geographic area], easy access. **(Coordination 06)**<br><br>*It was quite hard to get it started here…even though the project had been running elsewhere. There wasn't a really good general knowledge out there, certainly not in General Practices, or obstetric practices…and the number of people already offering RGCS was extremely limited.* **(Lead 04)**<br><br>*Something I wasn't really prepared for was kind of the lack of interest from health care professionals I really thought that we would kind of be fending them off and we are from certain pockets…but the areas where we really want higher recruitment from we're still not getting that at the moment which I don't know how we could have prepared for that but it would have been a good thing to consider before we launched.* **(Study GC 01)**<br><br>*If we think about how primary care is delivered in the Northern Territory with its scattered way…a very transient population, there are some very good GPs but waitlists for others are very, very, very, very, long. So difficult to see a GP to start off with.* **(Snr Member 01)**	
Tension for change	*It has been a bit of a dilemma, in terms of being unable to meet the needs of the general public but also knowing if you do push back and you are not the person who is able to see them, then there is some concern about the burden that is creating for other health care professionals.* **(Coordination 04)**	

	Somewhat frustrating…in terms of publicly funded there was very little we could offer...Even cystic fibrosis wasn't done as routine screening unless there was a particular reason to do it, like family history. **(Lead 05)**		
Compatibility	*Because [RGCS] is really complicated, and I think there was a lot of hesitancy from health care providers just feeling like they didn't really have enough training and they weren't qualified enough. They just didn't know how to integrate it into their practice. I think what we have seen is there have been some more willing to give it a go and they have built some momentum and there's been strong advocacy around making carrier screening accessible and over time others have jumped on the bandwagon.* **(Coordination 03)**<br><br>*Once GPs actually understood it's quite simple and probably only added 5 or 10 mins onto their consult. That again helped with their willingness.* **(Lead 05)**<br><br>*We had a little bit of kick back from some private obstetricians… their views on carrier screening and potentially feeling it is not in the best interest of their patients. They like everything to be happy…that they want the pregnancy experience to be a lovely experience and bringing up carrier screening and the potential of a difficult result, is not something they necessarily want to engage with. There has been increasing enthusiasm, but it hasn't been overwhelming.* **(Lead 04)**<br><br>*I think genetic counsellors are so well placed to undertake the counselling that is required for*	*I think that some of those [family history] questions could have been asked in a slightly different way or could have been tweaked to make it easier for the person reviewing it to know what action they need to take and when it is appropriate to follow-up.*[1] **(Coordination 04)**<br><br>*Family history follow-up…it's a lot. But the way you approach it is probably different for each [genetic] counsellor. The amount of follow-up they might do, or the amount of detail they might obtain probably varies. I'm a bit more of the model of, people are choosing to undertake this testing and therefore have a level of responsibility to get back to us when we ask for further information. So, I probably don't chase as much as the some of the other Genetic Counsellors.*[1] **(Coordination 05)**	*In terms of rolling out nationally, it's too labour intensive. So, you couldn't really do this if you did it for everybody, with the number of humans involved…you can't have a genetic counsellor that has to call people to follow-up on their family history. It's very nicely done, it's probably the best way to do it, but if you were actually going to do this as a population screen, it would have to be a lot more simplistic.* **(Lead 03)**

	RGCS…because we have experience in collecting family histories, understanding genetic risk, conveying that risk to couples, determining their concerns, their values, talking about their options and we have great comfort in discussing those things with couples, great experience and comfort. And being mindful about not putting our own values and beliefs on those conversations and not making judgements with couples, so that is something we do in our day-to-day work. **(Coordination 04)**		
Learning Climate	***The first 18-months of the study I spent a lot of time sharing my experience of how reproductive carrier screening programs worked and I feel like I wasn't really heard…I didn't really want us to reinvent the wheel because I felt like we had a really solid foundation of knowledge about how you translate this into the population.*** **(Coordination 03)**	Mackenzie's Mission is a research project; we will always be learning! (Variant Review Committee)<br><br>***All of our processes are the same as the rest of the Department...if any of the other people in the lab are running short, one of my team can fill in or if my team is short other members of the Department can fill in…at the analytical end we'd like to cycle people through so they can learn how to analyse samples diagnostically have a think about how you think about differently in a carrier setting.*** **(Laboratory 01)**	
Readiness for Implementation	***[The lab] has been doing these targeted panels for a very long time now. We know how they work and knew what we thought would happen.*** **(Lead 02)**<br><br>***So, we kind just went from one [screening program] and transitioned straight into the other. So, for me and my work I felt like everything was already set-up to go*** **(Coordination 02)**<br><br>***[The lab] was in a good position to develop Mackenzie's Mission in the sense that we [were] already [offering carrier screening]. So, in terms of***		

	carrier screening, report writing, having that relationship with their genetic counsellors and clinicians, that was already there.* (Laboratory 02)**<br><br>I think we felt confident as a unit that we could go ahead with this because of what it would likely mean for our unit. I don't think it was a big burden or anything like that.* (Snr Member 01)**		
Leadership engagement	***Because I am the director of the service and it is an area of interest, arguing the case to my team and it was unanimous support to say we can take this on.* (Lead 05)**<br><br>***I've had a long-standing interest in research aspects of screening and so my role is in oversight of the project…I was very involved in getting the guidelines through the College of Obstetricians…So very involved in screening for many years.*<br>(Lead 01)**<br><br>***Our role [leadership team] is we conceived the thing and wrote the grant application...and got the money to allow us to do this. And doing research into carrier screening has been a dream of mine for 30 years.* Lead 02)**		***We've got money for robots we've had that for nearly two years…if we receive those robots by the end of this year that would be considered pretty much a coup. It's new in the scale of it, and it's new in the style of what we're doing and the ability of the organisation to respond to that is not clear and because it's public service there's no one at the top who can say - look this needs to happen so let's just make it happen there's multiple levels of bureaucrats that go "Oh no I don't think that can happen so I'm putting a stop to it".* (Laboratory 01)**
Available Resources	***It was a well-resourced project, so an amazing grant from the government which was very exciting, and it was very much a matter of 'watch this space'* (Coordination 04)**<br><br>***The resources and the funding that was thrown at Mackenzie's Mission was phenomenal.*<br>(Coordination 06)**<br><br>***Health care professionals want to do this…they don't need an incentive. If we had taken that***	Budgeted FTE was insufficient to cope with sample load, and unevenly allocated between labs. (Laboratory Committee)<br><br>***I think we've been understaffed…particularly from the laboratory side of things. We didn't really have enough manpower to deliver on a four-week turnaround time.* (Coordination 01)**<br><br>***Our floor space is very limited so adding my team in, has caused a few crowding issues. If you think***	We need to double output by at least 6x (i.e. 2^6) to reach population scale! (Variant Review Committee)<br><br>The challenge of taking a research project straight into the real-world setting - i.e. when what has been a grant-funded service suddenly needs to be paid for by someone else. (Laboratory Committee)<br><br>Feedback during HCP interviews about the importance of Genetic Counsellors in education and recruitment, and implications for the MSAC

	money we had in the budget for trying to create a financial incentive for health professionals to offer this[2] and instead put it into genetics education, we would have got that done so much earlier and we would have created something to a really high standard. And we would have been able to tackle some of these issues around translated materials and some of the accessibility considerations in a much more productive way. **(Coordination 03)**	***about the scale of what we're doing here our department is about 75 people and they handle somewhere around 5,000 samples a year. The Mackenzie's Mission team is 5 people and the intention this year is people will handle about 5,000 samples so we're a bit busier than some of the others*** **(Laboratory 01)**	[Medical Services Advisory Committee] submission of Genetic Counsellor involvement in the future roll out of genetic screening. (Operational team)<br><br>***To run a proper national RGCS program you are going to need staff. You are going to need enough laboratory staff to be able to provide that screening in an appropriate timeframe. You are going to need dedicated genetic counsellors…who are now not just involved in recruiting but in all the aspect around people putting family history on forms all that sort of thing.*** **(Lead 04)**<br><br>***30,000 samples a year potentially, is 6 times larger than the entire department at the moment that's a pretty scary prospect and the only solution to that is going to be robotics to assist with the lab work and potentially bigger sequencers and so it involves spending money which is always in a publicly funded healthcare system a source of trouble and at that scale as well you think my God what are the space requirements that we would have? We'd need a much larger team and it's not clear that [the organisation] would immediately be able to say yes of course you need 10 or 15 new staff because it's all funded through Medicare funding even if we're bringing in Medicare funding the organisation does have these rules about how many people you're allowed to employ and it's not just if you've got money go ahead and employ someone so there's a lot of bureaucratic negotiation to be done to enable this to scale up fully***. **(Laboratory 01)**<br><br>***We always underestimate how many staff are going to be needed for the particular problem and***

			when you think about scaling up to that level you're asking staff to do what will ultimately be a highly repetitive task at a very high scale. **(Laboratory 01)**<br><br>***The number of people I have had to follow-up, and the number of test reports I have had to obtain to send off to the lab, things like that I don't think the GPs have the ability to do it. So, whether that is in the genetic service or if there is some kind of genetic counselling program available that goes along side this. For those with a family history, it's really difficult to apply population level RGCS to them. I think there definitely needs to be some kind of genetic counselling involvement*****.** **(Coordination 05)**<br><br>***Laboratory resources and their capacity. We have seen the pressure the laboratories get put under when you have pregnant couples enrolling and that does just put them under immense pressure. Also, the skills of the people in the laboratory and ability for them to be supported and have input from clinicians is really, really critical. The laboratory have mentioned the value they place on some of the family history things, so sometimes that is key.*** **(Coordination 04)**
Access to Knowledge & Information	Support and input from REDCap [research database] personnel from AGHA [Australian Genomics Health Alliance] has been crucial. (Research Committee)<br><br>***Situating the program within Australian Genomics was a huge benefit. So being able to tap into that infrastructure support, we would have been significantly delayed if we didn't have access to the REDCap support for example…and we had to start***	Expert opinions are worth their weight in gold. (Variant Review Committee)<br><br>***The thing about the review committee for Mackenzie's mission is we have 1300 genes, and they're across all sorts of different diseases. Nobody has all that expertise…And we have 30 to 40 people every week from around the country. And so far, it seems, no matter what gene has***	Beyond Mackenzie's Mission, we face the possibility of "knowledge drain" - labs are planning to maintain a small screening team until 3-gene and ECS [expanded carrier screening] become Medicare-funded. (Laboratory Committee)<br><br>Following up authors [who are knowledgeable on a specific variant] may not be practical at scale and

	***that from scratch, we would not be where we are today in this program - there's absolutely no doubt about it… having a program coordinator role and all that additional support around ethics approvals…has been really brilliant…and having that oversight from [the program manager] has really grounded the project and it meant it has been administered in a really professional way. I think that part of it has been really successful part of it.* (Coordination 03)**	***come up one of the clinical geneticists has seen a patient with that condition.*<br>(Lead 02)**<br><br>***I also like are our variant review committee, that meets every week, and being able to have that pool of knowledge to draw on is incredible.*<br>(Coordination 01)**<br><br>Input from Bioethics team valuable in development of the donor portal. (Research Committee)<br><br>***I think it's reassuring as well, to know that and to feel like this is the entire decision about whether a variant is pathogenic or not, is not entirely yours. There's other people who've looked at it and agree.* (Coordination 01)**	does not often change the final classification. (Variant Review Committee)<br><br>***Get a really good bioinformatician. Because they are worth their weight in gold. And they really understand both what you're trying to achieve but also the technical side of things*. (Coordination 01)**<br><br>***I think any lab that's going to get involved in this is going to have to have people that's experienced in doing the lab component. It would be very challenging to set up from scratch, there is a lot to learn, there's good guidance from RCPA [Royal College of Pathologists of Australasia] and NATA [National Association of Testing Authorities] on how you set up an accreditable next generation sequencing laboratory process.* (Laboratory 01)**<br><br>***Take advice from people who have done it before, who know how to take you through the accreditation barriers and who know the pitfalls. If you try and do it by yourself, you'll get there eventually but it will take you much longer and cost you much more.* (Laboratory 01)**<br><br>***Have a team of experts who have done this before to call on. Whether intimately involved in the set-up or not but having a group you can consult either on other various tasks or on the whole set-up. Don't reinvent the wheel.* (Coordination 02)**
**CFIR Domain** Characteristics of Individuals *involved in implementing a RGCS program*			
Knowledge & Beliefs about the Intervention	***It's been a welcome relief to be able to offer a couple a test that we know has value, where we know usually they would not normally be able to afford it.* (Lead 04)**	The tension between excluding a serious condition vs. giving false reassurance to couples that they've been screened for a condition. (Operational team)	***It should continue because I think there is great acceptability, and if you don't agree with it, you don't do it…it just gives people options*<br>(Lead 03)**

	Whilst it is often perceived as a complex topic, ultimately when you break it down it's pretty strong the argument for screening and that hasn't really shifted over time. **(Coordination 03)**	Sometimes there is no good outcome with variants of varying clinical consequence – to not report risks the couple having an affected child, to report risks and the couple terminating an unaffected pregnancy/pursuing IVF-PGT unnecessarily. (Variant Review Committee)<br><br>***It's not going to be perfect because it is screening, and it will miss things because it is screening. But it is important screening, and it does find quite a lot.*** **(Lead 03)**<br><br>***I think just accepting that this is a screening test, and it won't capture everything. That's been a big mantra. But it's really hard to do in practice, because you really want no one to be missed.*** **(Coordination 01)**<br><br>***In a screening setting there's so many genes that we are analysing and there's no phenotype, that you have to modify your criteria and there are a lot of times when we look at variants and we all take a deep intake of breath and go hmmm I'm worried about that one but it's not reportable and I just need to drop it and move on.*** **(Laboratory 01)**<br><br>***There are some genes in there that are tricky and potentially I guess, less severe than probably I would have ever expected…and some of those conditions can be really tricky for couples to make a decision about. It would be interesting to get some data from couples to see if they got results like that if they thought it was good information to receive or whether they thought it was more stressful.*** **(Study GC 01)**	***Mackenzie's Mission has been a good opportunity to explore a truly comprehensive, publicly funded test…and that it has been very successful and very well received by the public*** **(Lead 05)**<br><br>***I think it should be available to all. And I think it shouldn't depend on who you see or where you live. That shouldn't impact whether or not it is something that is available to you or offered to you and nor should it be if you can afford it or not.*** **(Coordination 04)**<br><br>***It's a great model, it's a great programme, it's been a pleasure to be involved with everybody on the team…this is exciting times in genetics and I think we need to really try and run with it and get it up and running properly*****. (Study GC 02)**

Self/collective efficacy	***For me it's fairly straightforward. I'm one of the originators of genomic sequencing in Australia. So, I've been doing this for years, and in terms of the actual laboratory work that's stuff I've overseen in my former laboratory and former life.***<br>**(Laboratory 01)**	VR committee becoming reasonably good at discussing and classifying variants. (Variant Review Committee)<br><br>The operational team is effective, listening well and finding solutions, especially given the complexities of the project. (Operational team)<br><br>The labs have done a fantastic job of it…they have gone more panel based, so they can go really high res and can see lots of little things. (Lead 03)<br><br>***Overall, the lab process itself does well and is really quite efficient, their biggest problem is that we haven't given them enough couples to actually scale up, but they have managed fantastically in the last few months when it has scaled up quite a bit.*** **(Coordination 02)**	
Individual Stage of Change	Evidence of clinicians making changes to their practice already (WA GPs have added discussion of RGCS/Mackenzie's Mission to their checklist for first trimester apt). (Education & Engagement Committee)	Capacity of lab team has increased, and morale is improving. (State team)<br><br>Common trend with few HCP recruiters doing much of the recruitment, and other HCPs recruiting occasionally. (Operational team)<br><br>***Certainly, the small number of GPs with an increased risk result that we have had, have definitely been converted to the value of carrier screening, because they can see how it can potentially help those couples*****. (Lead 04)**<br><br>***…they [HCPs] are in that same reproductive age group, so that's probably their motivation to offer this to other couples because they themselves would do it.*** **(Coordination 05)**	

Other Personal Attributes	***In hindsight, I understand why the project was rolled out initially in a few states and then became national over-time. Having said that, I think for me personally I would have preferred to have been involved from the very beginning, for various reasons. One, is that it can be hard to come on board to a project that is already full steam and to really feel like a fish out of water because you don't really know many of the details of the study and to also not have the opportunity to be involved in some of the elements of the study, so to not be able to give a perspective on some things has been a little frustrating as well.*** **(Coordination 04)**<br><br>***I would have liked to be involved from the start and I would have loved to be involved in some of the meetings in the lead-up to being launched and getting an understanding of – whose who and what's what? Because when we started… I'd get 10 different emails from 10 different people and my head was spinning.*** **(Coordination 06)**	Mackenzie's Mission Study team has been quite innovative to continue despite pandemic, not at a standstill which is great. (Research Committee)	
**CFIR Domain** Process: *of establishing and running a RGCS program*			
Planning	How large the project management plan is and how complex it is. (Research Committee)<br><br>We now have a plan in place in each of the 3 states about how to approach recruitment. (Recruitment Committee)<br><br>Concentrated on individual things that needs to be done. (Laboratory Committee)<br><br>More solid plans for the soft launch, including adequate piloting of data collection tools prior to the hard launch. (Research)	That the pandemic keeps throwing curve balls to the program. (Engagement)<br><br>Need to consider gradual transition to recruiting pregnant couples to avoid overwhelming labs. (Operational team)<br><br>Recruitment strategy – trying to slow recruitment overall and shift focus to under-represented areas. (State team)<br><br>Prioritising development of donor portal. (Psychosocial & Epidemiology Committee)	***We didn't spend any time in the first year thinking about the donor portal, translation into other languages and I get the reason why we didn't…but give yourself really good, strict deadlines to get things developed so you can get more of those ongoing things set-up as well. Take the time to get it set up as a whole service to minimise the amount of retrofitting you might need to do from the get go.*** **(Coordination 02)**<br><br>***Everything has to be thought through very carefully before you start such a program. Everything has to***

	Shifted from prioritising HCP contact, to development of documentation. (Recruitment Committee)<br><br>Good to be planning in advance for translation so this can be rolled out efficiently once content is finalised. (Education & Engagement Committee)<br><br>Plans for Aboriginal engagement progressing. (Executive Committee)<br><br>***I have also thought with any research project we should have included Aboriginal and Torres Strait Islander People at the start.* (Lead 03)**<br><br>Preparing a process to document drop outs/follow up with participants. (Operational team)<br><br>***My feeling was always that it was very well planned out…obviously drawing on pilot studies* (Coordination 04)**<br><br>***I felt like at the start of the study recruitment was really difficult and that was the big focus. But then we also weren't geared up enough in the lab to deal with the numbers that came through.* (Coordination 01)**	When there's time to plan and implement systems/processes properly, things work really well. (State team)<br><br>***It's hard to anticipate what is actually going to happen right?...and we could come up with as many plans as we like while everything is in theory and then we got to the real world and I guess figured that actually we had to change a lot of things but the good thing about Mackenzie's Mission was that it was so flexible and we could change things as we moved through.* (Study GC 01)**	***be in place before you start getting a flood of sample into the labs.* (Lead 04)**

Engaging	Upcoming meeting with support groups – starting to engage there. (Executive Committee)<br><br>Presence at GP education meetings and raising awareness of Mackenzie's Mission. (Recruitment Committee)<br><br>More engagement with Aboriginal community/leaders. (Recruitment Committee)<br><br>Recruiting of HCPs, cold-call of practices well received. (Education & Engagement Committee)<br><br>***It felt like there wasn't really a culture of health care professionals offering a lot of screening. So maybe the genetic counsellors could have done more through education sessions and maybe going a bit broader with our offer…in that early phase to get people on board and understanding what carrier screening is about.*** **(Coordination 01)**<br><br>***If you are calling because you want to engage them about something new that they don't have to engage in, then that's a very different situation...I think sometimes we don't even get to speak to those individuals because they don't respond.*** **(Coordination 04)**<br><br>***Finding the right person, motivating the manager. That's your first line, trying to convince them that it's a good idea. Sometimes that's tricky because they have no idea what genetic carrier screening is, and you sort of get the silence on the end of the phone. So being persistence*. (Coordination 06)**	Plan for education in remote/regional areas now relying more on videoconference, with the result that we can expand our geographic spread. (Education & Engagement Committee)<br><br>Indigenous Advisory Group established with state and territory representatives. (Clinical Committee)<br><br>Engaging participants and HCPs to read emails/welcome packs, etc. can be difficult. (Operational team)<br><br>Positive feedback about the HCP online education module. Module is serving a double purpose as a resource for existing HCPs. (Operational team)<br><br>Complexities in enrolling a same-sex couple, both of whom wish to have screening, and using the same donor. (Operational team)<br><br>***And it is very much more relationship building [in Aboriginal controlled health services]. You go and meet people…you've got to make the effort to go and see them.*** **(Lead 03)**	***If the question is about providing comprehensive, culturally safe and appropriate care in the Top End [of Australia], then it's about having structures in place and community engagement, and that is probably community by community. And the strength of the health service itself within that community.*** **(Snr Member 01)**<br><br>***We have Aboriginal Health care Practitioners who have a specific defined role within these primary health care facilities, and they have a certain defined scope of practice that is appropriate for their community and their remoteness. So, it could be, this is someone who is trained and from the community, knows their community best, and is best placed to communicate with their community about this [RGCS].*** **(Snr Member 01)**<br><br>***I think while we have been very conscious of appealing to the Aboriginal and Torres Strait Islander people…I think in practice it is going to be a lot harder than we think, I think we are almost going to need to train up Aboriginal health workers and have them go into community and give talks and spread the word and go to schools and really talk about this further. I'm not sure we are quite prepared for that at this point.*** **(Lead 05)**<br><br>***If all of this is funded, states will have to fund a team of Aboriginal health workers to go out into the community to spread the word, otherwise this isn't going to happen. I don't think there is enough opportunity for opportunistic discussion about this. Historically trying to delivery genetic services into the Aboriginal communities in the early days there was a lot of mistrust…I think it might be a good***

My tips and tricks are, if you are going to be emailing a HCP then you've got to be short, sharp and to the point…a lot of health care people just want 'Hi, this is it, are you onboard, yes or no?' It's really simple, it's a few lines…if you have got someone interested get back to them ASAP. Don't leave it a week to engage that person, it's almost an instant response. I also find giving people deadlines helps. Instead of just going 'can you get back to me'. (Coordination 06)		*opportunity to use this project to open up some doors.* (Lead 05)<br><br>*Hopefully the ability to do this will catch on and if it can be done in the setting we have chosen to target and it becomes successful and becomes available then that will filter through GPs to other GPs at the practices*. (Lead 05)<br><br>*I think it's about how do you engage with young individuals who are planning pregnancy or are already pregnant and that obviously to me is going to be the hardest part because we do miss people and there certainly is this window of young individuals who don't go and see their GP until they are pregnant so how do we capture that.*<br>(Lead 05)<br><br>*There's going to be groups and individuals within organisations who are going to be against this or are not comfortable with this sort of screening. Whether they are religious groups or same sex couples where this information is not going to apply to necessarily…and have we prepared ourselves or appropriately sat down with these groups to explain the purpose of the study or have we thought about will there be any strong vocal objection to this, so that would be one thing to consider.* (Lead 05)<br><br>*Somehow streamlining the HCP education would be great. So having webinars that are held every so often rather than ad hoc when someone is interested.* (Coordination 02)

			Education wise, I think that's a very easy thing to scale up…something like big webinars and some sort of certification that the doctors have to have in order to offer the tests would be much easier than obviously, individual genetic counsellors having to talk to individual practitioners.* (Coordination 01)**<br><br>I felt that sometimes in this project I am preaching to the converted… and it's really reaching out to all HCPs who would be relevant in raising this…how do we do that, whether it becomes a sort of mandatory thing, I don't know.* (Coordination 04)**
Opinion Leaders		***There are some people whose opinions you value more than others [at variant review committee meetings]. And we're going to lose that as people retire. One of the most impressive … [has] been doing metabolic procedure for decades. And she just knows it inside and backwards. So, if it's one of those that comes up, I'll go "what do you think?"* (Lead 02)**	
External Change Agents	The election result! We do know that we have ongoing support from the current health minister. (Recruitment Committee)		The changing tenure of the Health Minister is a risk to the future implementation of carrier screening. (Variant Review Committee)<br><br>***I think the big issue now is how this is going to be funded. I mean, [the current Health Minister] is really invested in this. He wants it to happen*. (Lead 02)**<br><br>***I think there is an appetite in Government, but as we know [the Health Minister] can't be in the job forever…* (Lead 03)**
Executing	Has been challenging to meet deadlines - a lot of material that needs to be put together, all happening in parallel. (Education & Engagement Committee)	***I think the 1300 genes has been very ambitious...We thought that most couples would have no variants that need looking at and would be able to be whipped through but in fact that's the***	***But I think the lab side really will need a lot of work to get that running to be able to do the numbers that are required and there's going to be a huge initial burst* (Lead 01)**

	Very difficult to develop everything and get it through review in the time we have. (Psychosocial & Epidemiology Committee)<br><br>First run and verification of NovaSeq went pretty well. (Laboratory Committee)<br><br>Same situation as NSW [New South Wales] facing system roadblocks in WA [Western Australia]. Flow on effects from when money is funded to actual recruitment takes a long time. (Laboratory Committee)<br><br>How quickly everything has come together over the last month to be ready to actually start testing, from feeling like it was impossible to now being ready to pilot. (Psychosocial & Epidemiology Committee)<br><br>Delay in HCP animation and deadlines slipping in general. (Education & Engagement Committee)<br><br>Everything is going as planned. (Research Committee)<br><br>Lack of recruitment in WA [Western Australia] due to couples already being pregnant: out of 9 HCPs, 3 managed to recruit. (Operational team)<br><br>[Consumer] instruction sheet gone really well by increasing compliance rate substantially. (Laboratory Committee)<br><br>Enrolment process shorter than expected a few weeks ago, ~40 mins. (Executive Committee)	***exception rather than the rule and so that has meant that the lab side has been harder than we thought.*** **(Lead 01)**<br><br>The low number of cases that have no variants to review (i.e., there is a lot of analysis). (State team)<br><br>Having samples in > reports out for a period earlier this year has led to an increase in TAT. This has negatively impacted recruitment of pregnant couples. (Laboratory Committee)<br><br>It can be tricky to strike the right balance between recruitment rate and lab capacity. Last year had strong recruitment but not enough lab capacity; now have the necessary lab capacity, but not enough recruitment. Aim is to get these two elements aligned. (State team)<br><br>Long turnaround time for results = many couples falling pregnant while waiting for results = more pregnant increased-chance couples. (State team)<br><br>Feel like we've achieved what we set out to do with Mackenzie's Mission: showed that the screening process works, we're identifying increased-risk couples and have recruited couples from all parts of Aus. Big tasks left to do: MSAC application & Aboriginal recruitment. (Executive Committee)<br><br>NSW [New South Wales] affected by lockdown in target regions and operating with split teams in the laboratory. (Operational team)<br><br>Amount of clinical time required to follow up [Family history]. (Operational team)	***I think consistency will be quite an issue…even within Mackenzie's Mission, there are differences between the labs and processes, and degrees of variant classification.*** **(Coordination 01)**

	***I think that [on-boarding HCPs] was well set up and that's because we have lots of genetic counsellors who were clinically thinking about how this would go, so I don't think there's been anything unexpected procedurally.* (Study GC 01)**	Learnt (and very surprised) that overall, the geographic spread of the Mackenzie's Mission cohort so far almost perfectly matches our national targets for each region. (Recruitment & Clinical Committee)<br><br>Learnt that different labs may take slightly different approaches to variant classification/reporting. (Variant Review Committee)<br><br>Variation between labs in terms of technical ability to detect variants means Mackenzie's Mission test is not truly 'national'. (Variant Review Committee)<br><br>***The lab has had a few where they've had to do re collections and so on, so that's also a process that we I guess set up but certainly it also takes time to re contact those people and people get frustrated as well having to do recollections.* (Study GC 01)**<br><br>***I thought we would have to do a lot more recollects from the cheek swabs than we've had to do.* (Lead 02)**<br><br>***Our recollect rate is lower than what we anticipated. And it's usually actually to do with one of our assays rather than the actual DNA extraction process*. (Laboratory 02)**<br><br>***I've been quite surprised that it has worked relatively smoothly. You know, there's always some couples who don't label things or don't include the test request form, but on the whole has been working well.* (Coordination 02)**

		So that's been kind of unexpected in how many errors there are. We've had people tick the same sex in their questionnaire, so then that breaks our LIMS (Lab Integration Management System). But the donor portal will obviously be a whole separate thing that we need to consider. **(Laboratory 02)**<br><br>***One thing that was unexpected, is the turn-around-time…it's getting more and not less. I think people have struggled with that.*** **(Coordination 06)**<br><br>***Our biggest problem is postage… postage can take 9 days there and 9 days back. That's 18 days delay, So, that has contributed to the turnaround times*****. (Coordination 06)**	
Reflecting & Evaluating	After the lab meeting, we have changed what data we will collect from the HCPs on couples they invite to the study. (Recruitment Committee)<br><br>Project moving forward in the right direction. (Laboratory Committee)<br><br>Flowcharts and spreadsheets that have been compiled are incredibly useful in understanding processes. (Research Committee)<br><br>We're alert to the issues & this committee is functioning well to raise issues. (Variant Review Committee)<br><br>The amount of effort involved in readying the HCP summary sheet when it seemed close to completion. (Education & Engagement Committee)<br><br>With the pilot we're able to sort out processes and workflow. (State team)	With recruitment underway, ideas to improve efficiency are becoming more apparent, e.g., portal improvements. (Operational team)<br><br>Proposed inter-lab data swap for all future increased chance results. (Variant Review Committee)<br><br>***To show that you can detect the same variants in people, you can swap DNA samples backwards and forwards. So, I think we've sent some samples across to [another lab] just to see that they can find the same variants that we do. But when you're doing 1300 genes, you're never going to know that you're going to get all of them. So as far as single nucleotide variants are concerted, you're certainly better off talking to the actual lab people.*** **(Lead 02)**<br><br>Different approach to recruitment of pregnant couples than the 'flick the switch' approach initially planned: geographically staggered and gradual	***I think the gene list is hard and the gene list needs to be regularly updated. Because that is the thing, as we find new stuff out, new genes will come onboard, other genes we thought were good we might end up finding are just a nightmare to interpret. So that will need to continue regardless as a committee because if you just leave it as it is it will become out of date quite quickly.*** **(Lead 03)**

	The pilot - received a lot of useful feedback which has led to changes to CTRL/REDCap [Dynamic consent form and Research database]. (Research Committee)<br><br>Feedback required from pilot study, but then costly to change things when portal already under development. (Operational team)	increase in eligibility cut-offs. (Laboratory Committee)<br><br>The wording of the reports requires greater flexibility to minimise confusion. (Variant Review Committee)<br><br>We are getting faster at reviewing variants but will need to get faster still and/or more stringent about which variants we spend time on as we scale up. (Variant Review Committee)<br><br>***I know that the gene review committee is meeting, they met earlier this year, we'll meet again next month to review some of the genes we flagged, should this be on the list? So, it's more as we go through it that we're realising.*** **(Laboratory 02)**<br><br>Clinical knowledge/literature can change rapidly, highlights importance on ongoing review of the gene list. (Gene Selection Committee)	

Notes:

[1] Family History information was self-reported by couples when they enrolled in the study. The question was deliberately broad to ensure as much relevant information was collected. Study genetic counsellors were to follow-up with couples if clarification was needed.

[2] In appreciation for their time, recruiting HCPs were given to option to claim a $35 reimbursement for each couple they saw that enrolled in the study and completed the evaluation survey at enrolment, irrespective of whether the couple went onto have screening or not.

[3] Participants who indicated that they were using an egg, sperm or embryo donor for their planned/current pregnancy were redirected to enrol via the Participant Donor Database. The enrolment process was largely the same as the standard enrolment process for couples with amendments made to facilitate enrolment and consent for everyone involved in a pregnancy using a donor.

[4] The study was progressively rolled out across Australia. Screening first began in four states/territories and continued in those states whilst being extended to the remaining states and territories.

Abbreviations: RGCS (Reproductive Genetic Carrier Screening); GP (General Practitioner, who fulfill a similar role to Family Physicians); HCP (Health Care Practitioner)
